# Proton-coupled alternating access in a versatile mycobacterial Spns drug transporter

**DOI:** 10.64898/2026.05.09.724020

**Published:** 2026-06-01

**Authors:** Samantha Gies, Kevin L. Jagessar, Tianqi Wu, Ian Miller, Khadijeh Dastvan, Reza Dastvan

**Affiliations:** 1 Department of Biochemistry and Molecular Biology, Saint Louis University School of Medicine, St. Louis, MO, USA; 2 Center for Applied AI for Protein Dynamics, Vanderbilt University, Nashville, TN, USA; 3 These authors contributed equally to this work.

## Abstract

Spns transporters are a mechanistically distinct branch of the major facilitator superfamily that regulate lipid transport, lysosomal homeostasis, immunity and disease, yet how the conserved Spns fold integrates protonation, substrate binding and alternating access to support chemically and directionally diverse transport activities remains unresolved. Here, we combine DEER spectroscopy in lipid nanodiscs with DEER- and AlphaFold-guided modeling to define the conformational landscape of the *Mycobacterium smegmatis* homolog *Ms*Spns. Protonation shifts *Ms*Spns toward an inward-facing state, whereas deprotonation favors a broader outward-facing ensemble through coordinated remodeling of intracellular and extracellular gates. These transitions are governed by membrane-embedded protonation switches and proton-sensing networks, while the substrate-binding cavity shows distinct proton sensitivity and weaker cooperativity. Hydrophilic cationic substrates, capreomycin and ethidium bromide, stabilize the outward-facing state, consistent with efflux antiport, whereas lipophilic compounds, including rifampicin, epicholesterol and selected phospholipids, favor the inward-facing state, suggesting uptake or allosteric stabilization. Thus, conserved proton-coupling elements can power opposing transport modes, revealing the mechanistic versatility of the Spns fold and its therapeutic potential.

Spns transporters have recently emerged as a mechanistically distinct and physiologically important branch of the major facilitator superfamily (MFS), with central roles in lipid transport, lysosomal homeostasis, immunity, and human disease^1–10^. The human Spns family comprises three lipid transporters, Spns1–3. Spns2 exports the bioactive lysolipid sphingosine-1-phosphate (S1P) from endothelial cells, thereby regulating S1P levels in circulatory fluids, lymphocyte trafficking, vascular function, and cell survival, and positioning Spns2 as a compelling immunomodulatory drug target^11^. Structural studies have rapidly transformed this field. Cryo-EM analyses captured human Spns2 in inward-facing (IF), occluded (O), and outward-facing (OF) conformations and revealed a noncanonical alternating-access cycle, while also defining the structural basis of inhibition^2^. Subsequent structures of Spns2 bound to S1P, inhibitors, and the immunomodulator FTY720-P further delineated the substrate-binding cavity, identified residues required for cargo recognition and conformational coupling, and provided an increasingly complete framework for transport and pharmacological modulation^3,4,6^. In addition, PI(4,5)P_2_ acts as a synergistic regulator that amplifies S1P-induced gate dynamics and transport^5^.

Progress on Spns1 has similarly broadened the biological scope of the family. Initially linked to lysosomal function and autophagic lysosome reformation, Spns1 is now recognized as a lysosomal lysophospholipid exporter that salvages phospholipids from the lysosomal lumen to the cytosol^8–10^. Functional studies established that Spns1 mediates the rate-limiting efflux of lysophosphatidylcholine (LPC) and lysophosphatidylethanolamine (LPE) and is essential for lysosomal lipid homeostasis^8^. This assignment is reinforced by mouse studies showing that Spns1 loss causes lysolipid accumulation and lysosomal storage disease-like phenotypes^9^. More recently, a cryo-EM structure of human Spns1 in an LPC-bound lumen-facing state identified structural features governing lysophospholipid recognition, revealed a TM5-TM8 luminal gate specialized for substrate entry, and uncovered a residue network implicated in proton sensing^10^. Consistent with this physiological importance, biallelic Spns1 loss-of-function variants cause a multiorgan disorder marked by liver and muscle injury, lysosomal lipid accumulation, and disrupted mTOR-regulated lipid homeostasis^12^. Together, these findings highlight the biomedical importance of defining how the Spns fold couples protonation, substrate chemistry, membrane lipids, and alternating access.

Despite these advances, key aspects of the transport mechanism remain unresolved, including energy coupling and how conserved Spns proton-coupling elements reshape the conformational landscape to enable chemically diverse and directionally distinct transport modes. Using an integrated approach in lipid membranes, we previously defined proton- and substrate-coupled conformational dynamics in the bacterial Spns homolog from *Hyphomonas neptunium* (*Hn*Spns)^7,13^. We identified conserved residues that regulate protonation and showed how sequential protonation of proton-switch residues coordinates conformational transitions. Consistent with this model, some recent studies support proton-coupled transport in Spns2^3^, whereas others propose alternative coupling mechanisms^2,14^ or emphasize substrate binding, conformational states, lipid regulation, or inhibition. Recent work on Spns1^8,10^ also strongly supports proton-coupled transport. Although the proposed luminal proton-sensing network in Spns1 is supported by mutational and cell-based transport studies, the contribution of membrane-embedded proton-sensing networks remains unexplored. Likewise, reverse mutation of the corresponding luminal network in Spns2 restores pH dependence of S1P transport^10^, but this does not exclude additional extracellular proton-sensing networks in Spns2 or other Spns proteins.

The bacterial Spns transporter MSMEG_3705 from *Mycobacterium smegmatis* (*Ms*Spns) provides a powerful system to address this gap by linking bacterial efflux and influx physiology to broader mechanistic principles governing lipid-transporting MFS proteins. *Ms*Spns has been implicated in selective drug resistance and broader mycobacterial physiology^7,15^. The structurally unrelated but hydrophilic antimicrobial compounds ethidium bromide and capreomycin, a cyclic polypeptide antituberculosis antibiotic, are substrates of *Ms*Spns, whereas the transporter does not confer resistance to the highly lipophilic first-line antituberculosis drug rifampicin^7,15^. Deletion of *Ms*Spns increases intracellular ethidium bromide accumulation, enhances capreomycin sensitivity, reduces rifampicin susceptibility, and accelerates growth in association with upregulation of the downstream isocitrate lyase gene^15^. These phenotypes suggest that *Ms*Spns couples selective efflux, possible uptake-like behavior, and adaptive mycobacterial physiology.

Spns proteins are secondary-active MFS transporters that use cation electrochemical gradients, most notably protons, to drive substrate transport^16–18^. Like other MFS transporters, they contain 12 transmembrane helices (TMs) arranged into two pseudosymmetrical six-helix domains, the N-terminal domain (NTD, TMs 1–6) and the C-terminal domain (CTD, TMs 7–12), which enclose a central substrate-binding and translocation cavity (Fig. 1a)^7,19,20^. These helices form four three-helix repeats, with TMs 1, 4, 7, and 10 lining the cavity, TMs 2, 5, 8, and 11 shaping the lateral gates, and TMs 3, 6, 9, and 12 providing structural support at the membrane interface^16^. The NTD is more strongly conserved across bacterial and eukaryotic Spns transporters, whereas the CTD is more divergent, suggesting a conserved mechanistic core superimposed on lineage-specific functional specialization (Figs. 1b and 1c)^7^. Residue maps of *Ms*Spns (Fig. 1b) and Spns2 (Fig. 1c) reveal clusters of highly conserved side chains within the transmembrane core, around the central cavity, and at putative gating interfaces, consistent with an extensive charge-relay and proton-coupling architecture. Defining how protonation remodels this network is therefore central to understanding alternating access across the Spns family.

Here, we use comprehensive double electron-electron resonance (DEER; also called PELDOR) spectroscopy^7,21–23^ in lipid nanodiscs, integrated with DEER- and AlphaFold-guided molecular modeling^24–27^, to define the conformational states of *Ms*Spns and determine how protonation, ligand chemistry, and membrane lipids bias its energy landscape. Our results establish the molecular basis for the dual behavior of this mycobacterial Spns transporter, including export of cationic hydrophilic compounds and uptake-like stabilization by lipophilic ligands. More broadly, they show how the conserved Spns fold can be adapted for bacterial efflux and influx while preserving core proton-coupling principles that connect *Ms*Spns to the emerging structural and dynamic framework of mammalian Spns transporters.

## Results

Mechanistic dissection of the Spns transport cycle requires defining the conformational states associated with substrate binding, translocation, and release, as well as the protonation switches that drive interconversion between these states. Because the cellular proton gradient is inwardly directed, *Ms*Spns could function either as a proton symporter that mediates substrate uptake or as an antiporter that couples proton influx to substrate efflux. To define the proton and ligand dependence of *Ms*Spns, which is presumed to couple inward proton translocation to substrate extrusion, we measured DEER distance distributions at pH 4 to favor protonation, at neutral pH 7.5, and at pH 9 to favor deprotonation. For selected reporter pairs spanning the substrate-binding cavity and gating helices, we also measured conformational responses to confirmed and putative substrates^7,15^. All experiments were performed with *Ms*Spns reconstituted into E. *coli* polar lipid nanodiscs.

### Structural and functional integrity of *Ms*Spns mutants

For DEER measurements, cysteine substitutions were introduced at selected sites on a cysteine-less (CL) background. We assessed the functional integrity of the CL construct and unlabeled double-cysteine mutants relative to wild-type (WT) *Ms*Spns using a previously established *Escherichia coli* cell-growth assay that reports resistance to toxic capreomycin concentrations and serves as a surrogate for *Ms*Spns-mediated drug efflux^28^. Consistent with our earlier findings that *Ms*Spns confers resistance to capreomycin and ethidium bromide, but not rifampicin^7,15^, cells expressing the CL and double-cysteine mutants survived capreomycin concentrations that were lethal to cells carrying the empty vector (Supplementary Fig. 1), confirming that the engineered constructs retained function.

As an additional probe of structural integrity, we monitored low-pH fluorescence quenching in spin-labeled *Ms*Spns reconstituted into lipid nanodiscs as a surrogate readout of conformational change (Supplementary Fig. 2)^29^. Protonation of WT *Ms*Spns at pH 4 reduced Trp fluorescence relative to pH 9, consistent with a protonation-dependent change in the local environment of Trp residues. Statistical analysis showed no significant differences between the spin-labeled double-cysteine constructs and CL *Ms*Spns, indicating that cysteine introduction and spin labeling at the probed sites did not measurably perturb this conformational response. Together, these assays support the structural and functional integrity of the reporter constructs, although direct effects of spin labeling on transport activity could not be assessed.

### Protonation promotes opening of the intracellular side

Motif A, the most conserved sequence motif in MFS transporters, lies in the intracellular loop between TM2 and TM3 (Fig. 2b)^16,30^. Together with surrounding residues from TM4 and TM11, it forms the conserved structural motif A, which stabilizes the O or OF state^16^. In Spns2 and Spns1, an associated charge-relay network (Asp163^TM2^-Arg167^TM3^-Asp220^TM4^ in Spns2) is proposed to regulate the interdomain charge-dipole interaction between TMs 2 and 11 (Fig. 2b). In mammalian Spns proteins, a symmetry-related motif A-like network is also formed by TMs 8, 9, 10, and 5 (Asp396^TM9^-Arg389^TM8^-Asp445^TM10^-Arg227^TM5^ in Spns2). These canonical motif A interactions are only partially conserved in *Ms*Spns. Backbone and side-chain hydrogen-bonding interactions between TM2 and TM11 (Asp84^TM2^-Ala357^TM11^/Thr358^TM11^) and between TM8 and TM5 (Asp288^TM8^-Ser147^TM5^/Ala148^TM5^) remain intact (Fig. 2a). However, *Ms*Spns uniquely lacks the highly conserved motif A aspartates on TMs 4 and 10 and therefore cannot form the corresponding interdomain salt bridges with Arg356^TM11^ and Arg146^TM5^, respectively (Fig. 2b). In the AlphaFold3-derived OF model of *Ms*Spns, these missing interactions appear to be compensated by an alternative Arg356^TM11^:Glu426^TM12^:Arg146^TM5^ salt-bridge network (Fig. 2a).

Upon *Ms*Spns deprotonation at pH 9, the intracellular TM2/TM11 and TM5/TM8 gates become highly dynamic (Fig. 2c). Deprotonation shifts the two gate ensembles in opposite directions, favoring a closed state for TM5/TM8 and an open state for TM2/TM11, although a closed TM2/TM11 population remains detectable at pH 7.5. Protonation at pH 4 reverses these shifts. Importantly, protonation does not close the TM2/TM11 gate as predicted by the OF model; instead, it favors an IF arrangement and promotes opening of the intracellular side.

The proton-dependent distance changes observed for reporter pairs on the cavity helices (Fig. 3) closely mirror those of the gating helices. The dominant deprotonated and protonated intermediates match the distances predicted by the OF and IF models, respectively, supporting a protonation-driven shift toward intracellular opening. By contrast, the W22-N140 pair, which reports on TM1-TM4 intradomain flexibility, shows no detectable protonation-dependent change, consistent with a predicted Asp11^TM1^-Arg139^TM4^ salt bridge that may constrain local TM1-TM4 motion.

Distance pairs involving the support helices TMs 3, 6, 9, and 12 (Figs. 4a and 4b) follow the same global proton-dependent transition observed for the gating helices. In the deprotonated state, these support helices sample broader conformational ensembles, whereas protonation shifts the equilibria toward shorter, IF-like distances. For the A90–L298 and L197–L298 distance pairs (TM3/6–TM9), the OF state is only marginally populated under deprotonated conditions. By contrast, intradomain support-helix pairs (A90-L197, I230-L298, I230-R421, W22-A90, W22-L197, and L298-L352; Fig. 4 and Supplementary Fig. 3) show minimal protonation-dependent changes, partially supporting a rocker-switch-like transition between IF and OF states. Two conserved CTD intradomain interactions in mycobacterial Spns proteins^15^, a salt bridge between Asp294^TM9^ and Arg421^TM12^ and a hydrogen bond between Ser227^TM7^ and Asp423^TM12^ (Fig. 4a), remain intact throughout these transitions, as indicated by the narrow, proton-independent distance distributions of the corresponding reporter pairs (Fig. 4c).

### Protonation promotes closure of the extracellular side

On the extracellular, cell-envelope side, the salt bridge between an aspartate on TM2 and an arginine on TM7 (Asp57^TM2^:Arg255^TM7^ in *Ms*Spns; Fig. 5a) is among the most conserved signature interactions in bacterial and mammalian Spns transporters. Together with additional interacting residues, including a highly conserved aspartate on TM11, this interface forms an extracellular proton-sensing network proposed to drive the IF-to-OF transition (Figs. 5a and 5b). Variation in the participating residues and interaction types likely contributes to mechanistic diversity across the family^10^. For example, in human Spns3, which lacks the TM7 arginine, the equivalent salt bridge may instead be formed by the preceding lysine residue (Fig. 5a).

Under acidic conditions, protonation closes the extracellular TM2/TM11 and TM5/TM8 gating pairs (Fig. 5c). As on the intracellular side, the deprotonated state is most consistent with a broad OF-like ensemble, indicating disruption of the proton-sensing network. Notably, for the TM5/TM8 distance pair A172-M260, the average distance is shorter in the deprotonated state despite the OF conformation. In addition, unlike the pronounced opening of TM2/TM11, opening of the TM5/TM8 gate is modest during the IF-to-OF transition (Figs. 5b and 5c).

Reporter pairs spanning the cavity helices and proton-sensing network show the same proton-dependent remodeling (Fig. 6). The E54-M254 pair, which directly monitors the highly conserved TM2-TM7 salt bridge, shows that deprotonation disrupts this interaction and stabilizes the OF state. Additional pairs spanning TM2/TM8, TM5/TM7, and TM6/TM7 support this transition, revealing an extensive extracellular proton-sensing network across TM1/TM2, TM5/TM6, TM7/TM8, and TM11. For pairs linking TM4 with TM7/8 cavity helices (S111-M254/S260; Fig. 6b), protonation stabilizes a long-distance intermediate that is absent from the IF state and only weakly sampled in the OF state. Nevertheless, consistent with the overall trend, the OF state remains dominant under deprotonated conditions for these pairs. As predicted by the models, the intradomain E54-A108 pair shows only marginal changes.

Distance pairs involving extracellular support helices likewise follow the global protonation-dependent OF-to-IF transition (Fig. 7).

### Substrate-binding cavity of *Ms*Spns senses the proton differently

We next examined the pH-dependent conformational equilibrium of ligand-free *Ms*Spns in lipid nanodiscs using intracellular and extracellular reporter pairs. Changes in the population of the increasing state(s) as a function of pH were used to estimate the apparent pK of the conformational transition (Fig. 8). Nonlinear least-squares fitting yielded basic pK values of 7.7± 0.0 (*n* ≈ 2.5±0.7) and 8.0±0.1 (*n* ≈ 1.7±0.7) for the intracellular pairs A90^TM3^-L298^TM9^ and R145^TM5^-L290^TM8^, which report on support- and gating-helix motions, respectively (Figs. 8c and 8d). Because this apparent pK reflects protonation/deprotonation of acidic residue(s) that drive *Ms*Spns isomerization, the elevated values likely arise from buried sites in the central cavity, such as Asp38^TM1^ (Fig. 8a), and from motif A-associated hydrogen-bonding interactions between Asp84^TM2^ and Ala357/Thr358^TM11^ and between Asp288^TM8^ and Ala148/Ser147^TM5^ (Fig. 8b, inset). These interactions are expected to be stronger when the aspartates are deprotonated and act as hydrogen-bond acceptors, consistent with deprotonation favoring intracellular closure. The steep apparent Hill coefficients indicate a highly cooperative, switch-like transition, suggesting that multiple protonation/deprotonation events are energetically coupled into a concerted macroscopic response rather than acting independently.

By contrast, the intracellular N140^TM4^-I230^TM7^ pair, which reports on relative motion of the substrate-binding cavity helices, yielded a lower pK of 6.8±0.1 (*n* ≈ 0.7±0.1) (Figs. 8e and 8f). This more acidic pK likely reflects the lower proton affinity of the less buried putative protonation switch Glu126^TM4^ (Figs. 8a and 8b). A Hill coefficient near 1 indicates that the conformational transition of the cavity helices is weakly cooperative and may arise from more independent protonation events.

Accordingly, on the extracellular side, the T179^TM6^-M254^TM7^ pair shows a similarly basic apparent pK of 7.8±0.0 and cooperativity of 1.9±0.2, closely matching the intracellular gating/support helices (Figs. 8g and 8h). This behavior is consistent with the structural connectivity of TM5/TM6 and TM7/TM8 at the extracellular gate. Likewise, the E54^TM1/2^-M254^TM7/8^ cavity-helix pair, which monitors the conserved Asp57^TM2^:Arg255^TM7^ salt bridge, exhibits an elevated pK of 7.5±0.1 because it is coupled to the gating helices. However, its Hill coefficient of approximately 1 suggests a more independent protonation response, consistent with the behavior of the substrate-binding and release helices.

Interestingly, the extracellular pairs E54^TM1/2^-S384^TM11^ and E54^TM1/2^-A108^TM3^ display biphasic pH titration behavior, consistent with two sequential protonation-linked conformational transitions (Figs. 8i and 8j). This behavior is plausible given the extensive extracellular proton-sensing network and its coupling to membrane-embedded protonation switches. For E54-S384, which directly reports on TM2/TM11 gating and is sensitive to the hydrogen-bonding interaction between Asp383^TM11^ and Asp57/Thr58^TM2^, a basic pK of 7.9±0.1 (*n* ≈ 1.1±0.2) is observed for the state corresponding to complete gate disruption (Fig. 8i), consistent with the global gating transition.

The E54-A108 pair monitors the proposed proton-transfer pathway to Asp38^TM1^ through highly conserved Spns residues (Figs. 8a and 8b), including Trp99 and Thr103 on TM3 and Trp177 on TM6^7^. We previously proposed a conserved Spns proton-coupling mechanism in which protonation and deprotonation of the TM4 glutamate are regulated by the protonation state of the TM1 aspartate and by substrate binding^7^. Our extensive MD simulations further implicated neutralization of the highly conserved TM4 and TM1 arginine residues in this regulatory mechanism (Fig. 8a)^7^. Consistent with this model, the AlphaFold3-generated IF structure shows deprotonated Asp38^TM1^ forming a salt bridge with Arg119^TM4^ and a hydrogen bond with Thr103^TM3^ (Fig. 8b, IF). This arrangement is expected to favor protonation of Glu126^TM4^ and disruption of its salt bridge with Arg39^TM1^. In the OF model, by contrast, the Asp38^TM1^ interactions are disrupted (Fig. 8b, OF), potentially mimicking Asp38 protonation. Thus, Asp38-linked conformational changes can be transmitted directly to the extensive extracellular proton-sensing network through interactions involving Glu47, Lys50, Asp52, Asp57, and Thr58 (Fig. 8b, inset).

### Substrate hydrophobicity drives opposing shifts in *Ms*Spns conformational equilibria

Deletion of *Ms*Spns in *Mycobacterium smegmatis* increases intracellular accumulation of, and sensitivity to, water-soluble compounds such as capreomycin and ethidium bromide, while reducing susceptibility to lipophilic drugs such as rifampicin^7,15^. Consistent with an efflux antiporter mechanism, the cationic compounds capreomycin and ethidium bromide stabilize the OF conformation at pH 7.5 relative to the apo state (Fig. 9 and Supplementary Figs. 4 and 5). Docking capreomycin into the IF conformation highlights highly conserved aromatic residues across the binding cavity of mycobacterial Spns proteins that may contribute to substrate binding (Fig. 9a). By contrast, lipophilic compounds, including rifampicin, shift the conformational equilibrium toward the IF state, consistent with possible uptake. Previous studies showed that the lipophilic Spns2 inhibitors 16d (SLF1081851) and 33p (SLB1122168) attenuate transport by trapping Spns2 in an inward-facing state^2,6^.

Loss of *Ms*Spns was previously shown to strongly upregulate the downstream gene encoding isocitrate lyase (ICL)^15^. Defective efflux may increase intracellular accumulation of xenobiotics and metabolic by-products, thereby triggering compensatory metabolic responses. In *Mycobacterium tuberculosis,* ICL is broadly induced during antibiotic tolerance and persistence^31^, suggesting that *Ms*Spns-linked changes in ICL expression may reflect a broader stress-adaptation program. The genomic linkage of a 3-α-hydroxysteroid dehydrogenase (MSMEG_3704), *Ms*Spns (MSMEG_3705), and an ICL (MSMEG_3706) suggests a functional connection to lipid or sterol catabolism, consistent with the role of ICL in buffering acetyl- and propionyl-CoA flux. Although *Ms*Spns confers resistance to cationic antibiotics such as capreomycin, its native substrates may include cationic amphipathic molecules, sterol-derived catabolites, or other amphipathic metabolites. Consistent with this possibility, cholest-5-en-3α-ol (epicholesterol), a sterol derivative and potential lipophilic substrate, stabilizes the IF conformation, like rifampicin, based on the E54-M254 and R145-I230 distance pairs (Fig. 9 and Supplementary Figs. 4 and 5), suggesting uptake.

This dual behavior, together with the opposing effects of water-soluble and lipophilic compounds on conformational equilibria, parallels our findings for *Hn*Spns^7^ and may represent a shared mechanistic signature of Spns proteins.

Interestingly, the lipid composition used in the *Hn*Spns study also shifts *Ms*Spns toward the IF state across pH values (Fig. 9d and Supplementary Fig. 6), while preserving the differential effects of hydrophilic and lipophilic substrates (Fig. 9 and Supplementary Fig. 5). This finding suggests that specific lipid environments can tune the conformational landscape without overriding ligand-dependent directionality. Because phosphatidylinositol (PI) binds Spns2 with relatively high affinity among non-phosphorylated glycerophospholipids, possibly reflecting a broader preference for inositol-containing or anionic lipids in Spns proteins^5^, L-α-phosphatidylinositol (SAPI) in this lipid mixture could act as a potential *Ms*Spns substrate or allosteric modulator that stabilizes the IF conformation, similar to rifampicin or epicholesterol. The IF stabilization by PI is particularly intriguing because mycobacterial membranes contain phosphatidylinositol, an essential precursor for phosphatidylinositol mannoside, lipomannan, and lipoarabinomannan biosynthesis, which shape cell-envelope architecture and host-pathogen interactions^32,33^. Accordingly, the recent observation that Spns2 exports S1P while importing glucose^14^ supports the broader possibility that Spns transporters can couple movement of chemically distinct ligands in opposite directions.

### DEER-guided refinement and modeling of IF and OF *Ms*Spns structures

To convert the experimental DEER data into structural models, we used DEERFold^24^, a fine-tuned AlphaFold2-based network^26^ that incorporates DEER distance distributions as distogram inputs to predict spin-label-constrained conformations. Single-Gaussian approximations of the IF- and OF-dominant distance distributions measured at pH 4.0 and pH 9.0, respectively, were used as DEERFold restraints. The resulting IF and OF ensembles closely matched the corresponding unbiased AlphaFold3 (AF3) models (Fig. 10a)^27^. The top IF models showed an average TM-score of 0.96 and an average Cα RMSD of 1.86 Å relative to the AF3 IF model, whereas the top OF models showed an average TM-score of 0.97 and an average Cα RMSD of 1.76 Å relative to the AF3 OF model. Refinement of the unbiased AF3 models using the same DEER distance constraints produced similarly convergent structures (Fig. 10a and Supplementary Figs. 7 and 8)^25^. When the complete multi-Gaussian distance distributions from all three pH conditions were used, DEERFold generated more dispersed ensembles, consistent with its fine-tuning on single-Gaussian distance distributions^24^. The best pH 4.0 model reached a TM-score of 0.86 and a Cα RMSD of 3.61 Å relative to the AF3 IF model, whereas the best pH 9.0 model reached a TM-score of 0.86 and a Cα RMSD of 3.56 Å relative to the AF3 OF model (Fig. 10a). Even with this lower-precision representation, the pH-dependent trend remained clear: protonated restraints at pH 4.0 produced more IF-like models, whereas deprotonated restraints at pH 9.0 produced more OF-like models, demonstrating that the IF-to-OF conformational shift is robust across distance-restraint representations.

Tunnel analysis of the unbiased and refined AF3 models, together with the top DEERFold models (dark green; Figs. 10b and 10c), indicates that in the IF state the substrate-binding site is accessible laterally from the inner membrane leaflet through the TM2/11 and TM5/8 gates and directly from the cytoplasm. Thus, both lipophilic and water-soluble substrates can enter or exit the transporter in this state (Fig. 10b). In the OF state, the extracellular TM5/8 membrane-facing gate is constricted, and substrates can access or leave the central cavity through the lateral TM2/11 gate or directly from the periplasm (Fig. 10c).

## Discussion

Using an integrated spectroscopic and computational approach, we define the proton- and substrate-coupled alternating-access mechanism of *Ms*Spns and show how a conserved Spns fold supports mechanistic versatility. DEER spectroscopy in lipid nanodiscs, combined with DEER-guided and AlphaFold-based modeling, reveals that protonation shifts *Ms*Spns toward an IF state, whereas deprotonation favors a broader OF ensemble through coordinated remodeling of the intracellular and extracellular gates. These transitions are governed by an extensive proton-sensing architecture composed of membrane-embedded protonation switches and an extracellular proton-sensing network, while the substrate-binding cavity displays distinct proton affinity and weaker cooperativity, indicating differential proton sensing within the transporter. Functionally, hydrophilic cationic substrates such as capreomycin and ethidium bromide stabilize the OF state, consistent with efflux antiport, whereas lipophilic compounds such as rifampicin, epicholesterol, and selected phospholipids shift the equilibrium toward the IF state, suggesting uptake-like transport or allosteric stabilization. Together, these findings establish *Ms*Spns as a proton-coupled transporter with opposing responses to chemically distinct substrates and provide a structural framework that connects bacterial efflux and influx to the emerging mechanistic landscape of mammalian Spns proteins.

The proton-coupled ensemble shifts observed here are substantial, internally consistent across many reporter sites, and well supported by both DEER-guided and unbiased structural models. This convergence strengthens the conclusion that *Ms*Spns does not simply toggle between two static structures, but instead samples a proton-dependent energy landscape in which gating helices, support helices, and the substrate-binding cavity are differentially coupled. Future mutational and transport studies of the conserved residues identified here should define their energetic contributions to alternating access and substrate directionality.

Based on the AF3 and DEER-refined IF and OF models of *Ms*Spns, together with its proton- and ligand-dependent conformational dynamics, we propose a dual transport model for *Ms*Spns (Figs. 10d and 10e). We previously proposed for *Hn*Spns a proton-coupling mechanism centered on highly conserved membrane-embedded protonation switches (Fig. 8a), suggesting that this mechanism is broadly conserved across the Spns family^7^. This model identifies residues critical for protonation and its regulation, explains how sequential protonation of these switches drives conformational transitions, and provides a mechanism for coupling proton movement to substrate transport, including a putative proton-translocation pathway (Fig. 10). In this model, the resting state is OF (Fig. 10d, steps 1 and 4), stabilized by protonation of Asp38^TM1^, likely through a periplasmic tunnel leading to Asp38 (Fig. 10c, lime tunnel in the AF3-refined model) and involving conserved Spns residues, including Trp99 and Thr103 on TM3 and Trp177 on TM6 (Fig. 10d, step 4)^7^. The IF state (steps 2 and 3) represents a higher-energy conformation and is stabilized by protonation of Glu126^TM4^. During the OF-to-IF transition (Fig. 10d, step 1 to 2), periplasmic release of a cationic water-soluble substrate such as capreomycin primes Glu126 for protonation from the periplasmic side, while proton transfer from Asp38 to acidic residues at the intracellular gates, such as Asp84, disrupts stabilizing hydrogen bonds, favors the IF conformation^34^, and primes the transporter for substrate binding from the intracellular side (step 2). In this state, deprotonated Asp38 can form a salt bridge with Arg119^TM4^ and a hydrogen bond with Thr103^TM3^. Binding of cationic substrates then induces deprotonation of Glu126, either through proton transfer to the intracellular side or relay back to Asp38, followed by salt-bridge formation with Arg39^TM1^ (step 3). Capreomycin binding together with Asp38 protonation generates local conformational changes that propagate to the extracellular proton-sensing network, stabilizing the OF state (step 3 to 4), consistent with antiport. Notably, capreomycin, a polypeptide antibiotic, behaves as a tetraprotic acid and can also serve as a proton donor at pH 7.5^35^. Four intradomain salt bridges and hydrogen bonds (Lys50^TM1^:Asp57^TM2^, Glu267^TM8^:Arg322^TM10^, Asp294^TM9^:Arg421^TM12^, and Ser227^TM7^-Asp423^TM12^) remain intact during IF-to-OF isomerization (Fig. 10d, steps 3 and 4), consistent with the proton- and substrate-independent behavior of most intradomain DEER distances (Figs. 3, 4c, 6, and Supplementary Fig. 3b). By contrast, binding of lipophilic substrates such as rifampicin directly from the outer membrane leaflet, together with Glu126 protonation, drives OF-to-IF isomerization and release of protons to the cytoplasm and substrate to the inner leaflet, consistent with symport-like uptake (Fig. 10e).

**Figure 1.**
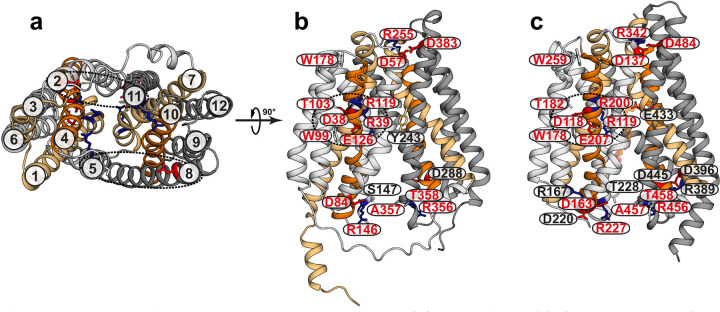
Domain architecture and conserved residues of Spns proteins. (**a**) Cytoplasmic view of the inward-facing (IF) *Ms*Spns model, with the N-terminal domain (NTD) and C-terminal domain (CTD) shown in light and dark gray, respectively. (**b**) Membrane view of *Ms*Spns highlighting conserved Spns-family residues in red and *Ms*Spns-specific functional residues in black. Symmetry-related TM helices 1/7 and 4/10 are colored light and dark orange, respectively. (**c**) Cryo-EM structure of human Spns2 bound to S1P ((Protein Data Bank (PDB) code 8EX4), with functionally conserved residues in mammalian Spns proteins labeled in black.

**Figure 2.**
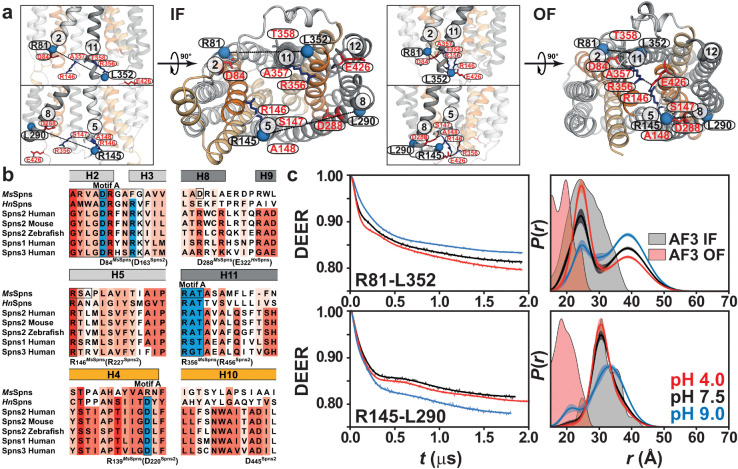
Proton binding promotes opening of the intracellular gates in *Ms*Spns. (**a**) Spin-label pairs (blue spheres) used for DEER distance measurements across intracellular gating helices in the NTD and CTD of IF and OF *Ms*Spns models. Membrane-view insets show the corresponding gate architecture. Functional residue interactions that stabilize the OF conformation are highlighted in red. (**b**) Sequence alignment of Spns proteins and bacterial homologs, with residues shaded by conservation in red. Residues forming structural motif A are shown in blue, and *Ms*Spns residues that stabilize the OF state are boxed. (**c**) Raw DEER decays and fits (left) and the corresponding distance distributions, *P*(*r*), measured in nanodiscs for R81–L352 and R145–L290 under acidic, neutral, and basic pH conditions. Confidence bands (2σ) are shown about the best fit lines. This band, which depicts the estimated uncertainty in *P*(*r*), reflects error associated with the fitting of the primary DEER trace. Distance distributions predicted from the IF and OF models are shaded gray and red, respectively.

**Figure 3.**
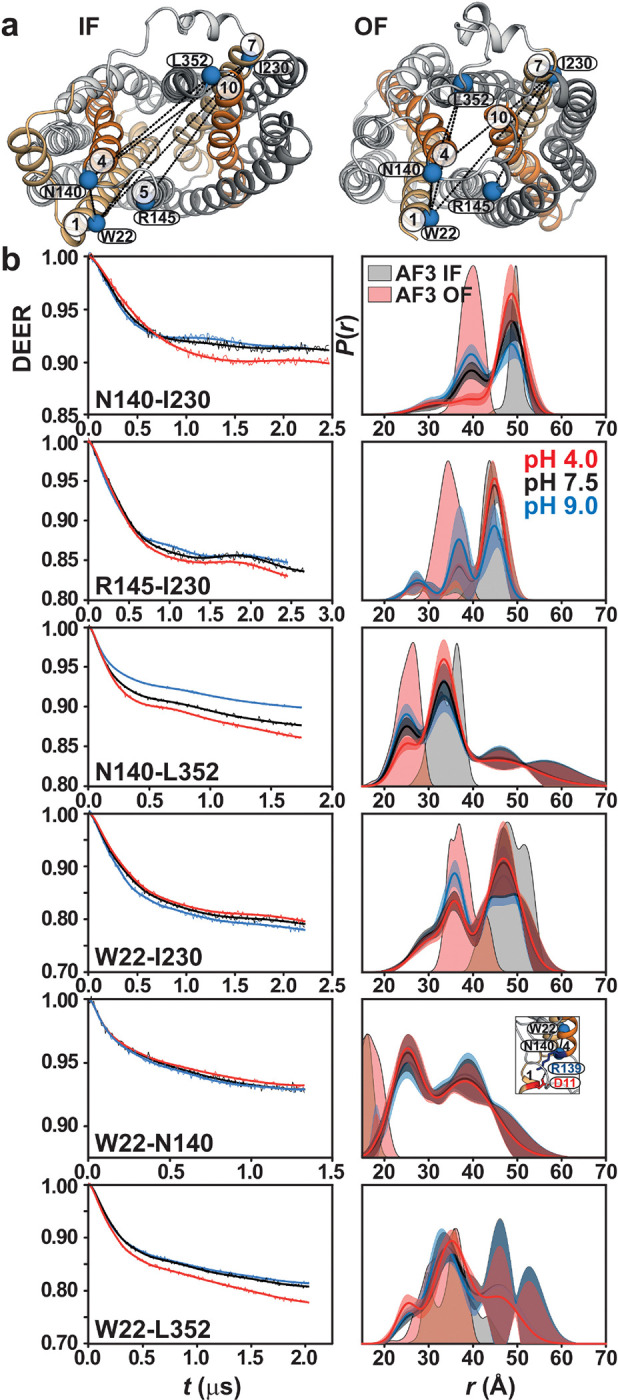
Protonation promotes intracellular opening of the substrate-binding cavity. (**a**) Spin-label pairs (blue spheres) reporting on cavity-associated helices are shown on the intracellular side of the IF and OF models. (**b**) Raw DEER decays and fits (left), together with the corresponding distance distributions, *P*(*r*) (right), measured in nanodiscs for the indicated spin-label pairs under acidic, neutral, and basic pH conditions. Distance distributions predicted from the IF and OF models are shown as gray and red shaded regions, respectively.

**Figure 4.**
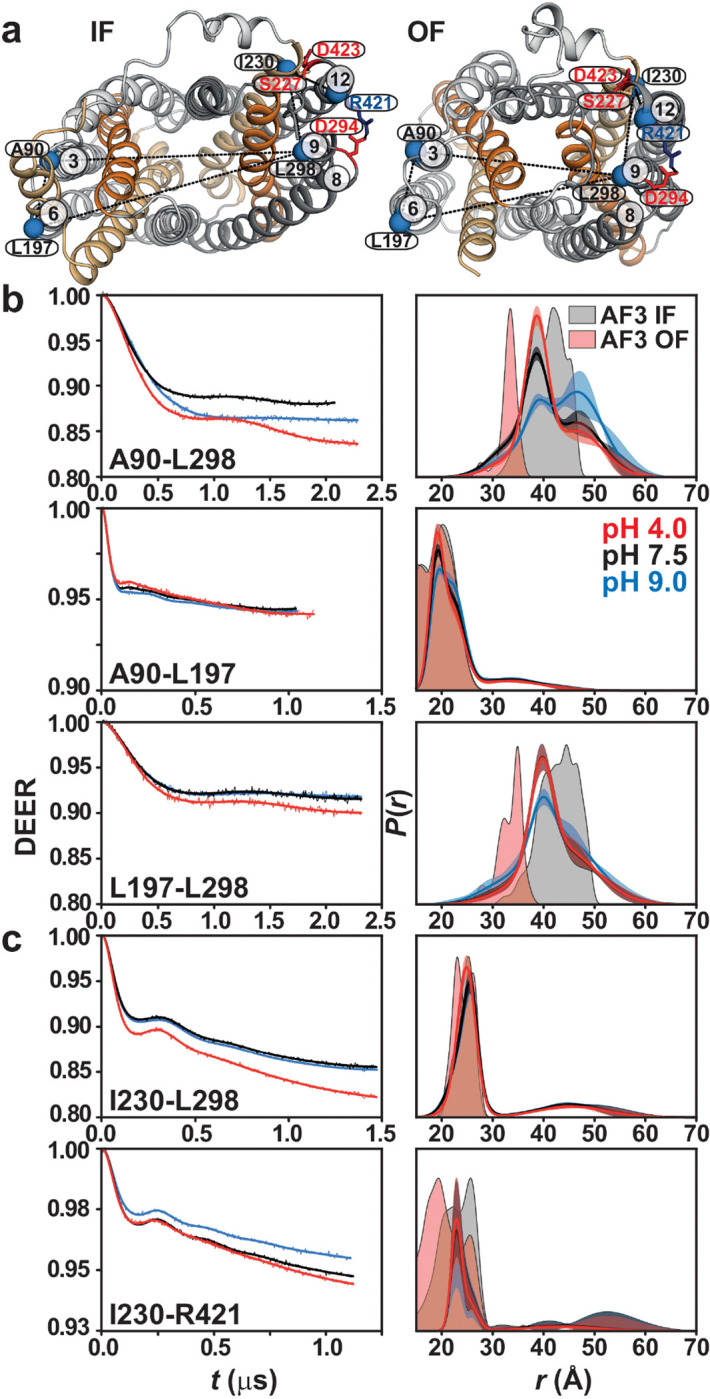
Protonation rearranges support helices while preserving an IF-like state. (**a**) Spin-label pairs reporting on the support helices are shown as blue spheres on the intracellular side of the IF and OF models. Intradomain interacting residues are shown as sticks. (**b**) Raw DEER decays with fits (left) and corresponding distance distributions, *P*(*r*) (right). (**c**) Distance pairs reporting on intradomain salt-bridge and hydrogen-bond interactions. Distance distributions predicted from the IF and OF models are shown as gray and red shaded regions, respectively.

**Figure 5.**
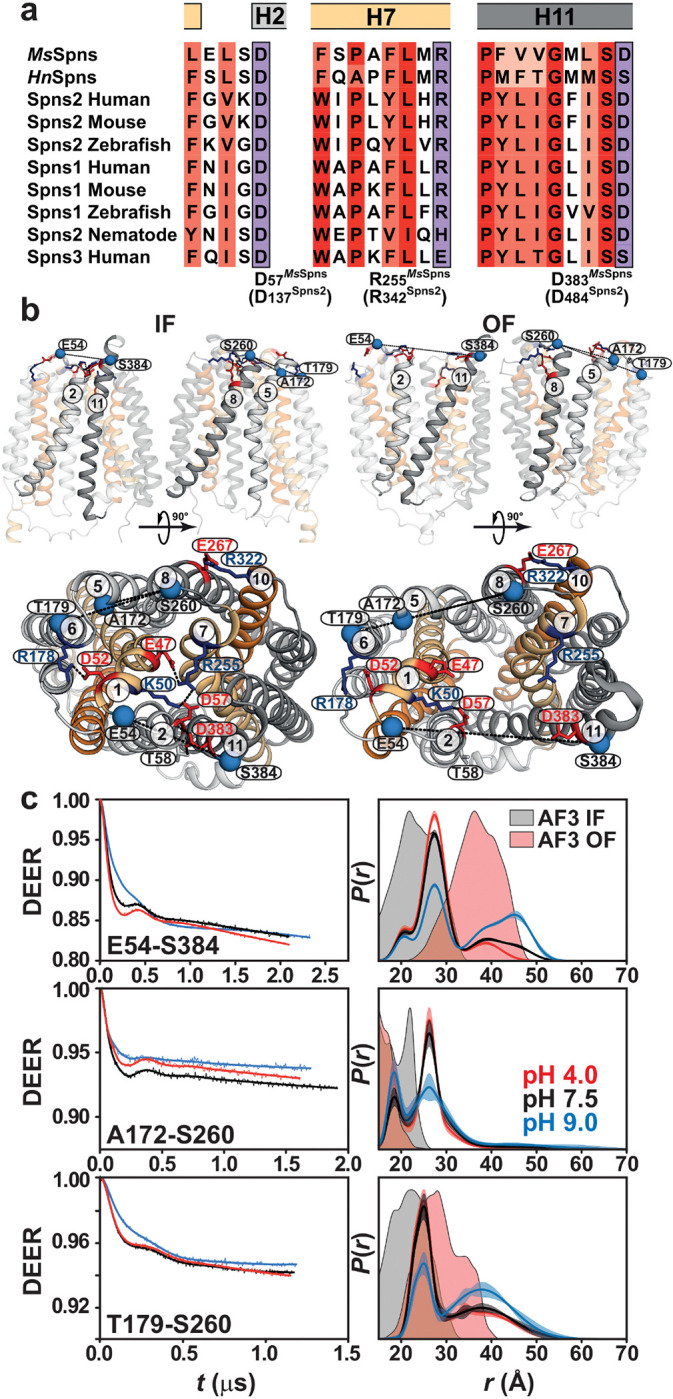
Proton binding promotes closure of the extracellular gates. (**a**) Sequence alignment highlighting conserved elements of the extracellular proton-sensing network, shaded in purple. (**b**) Membrane views of the IF and OF models show the architecture of the extracellular gates. Periplasmic views of the OF state show the proton-sensing network as sticks, with DEER spin-label pairs indicated as blue spheres. (**c**) Raw DEER decays with fits (left) and corresponding distance distributions, *P*(*r*) (right), for E54–S384, A172–S260, and T179–S260. Distance distributions predicted from the IF and OF models are shown as gray and red shaded regions, respectively.

**Figure 6.**
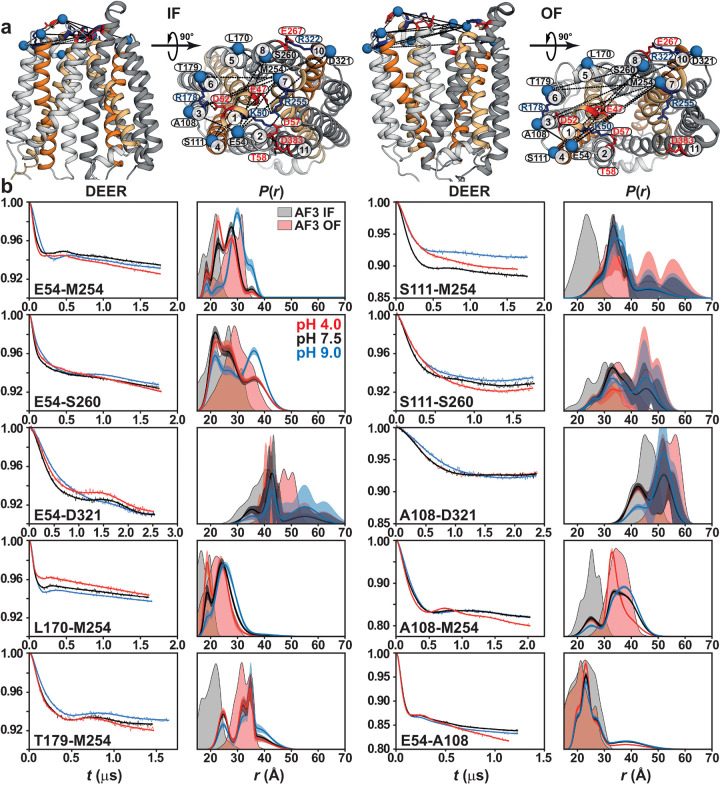
Protonation reshapes the proton-sensing network to close the extracellular side. (**a**) Spin-label pairs reporting on the extracellular proton-sensing network and binding cavity are shown as blue spheres on the IF and OF models. (**b**) Raw DEER decays with fits and corresponding distance distributions, *P*(*r*). Distance distributions predicted from the IF and OF models are shown as gray and red shaded regions, respectively.

**Figure 7.**
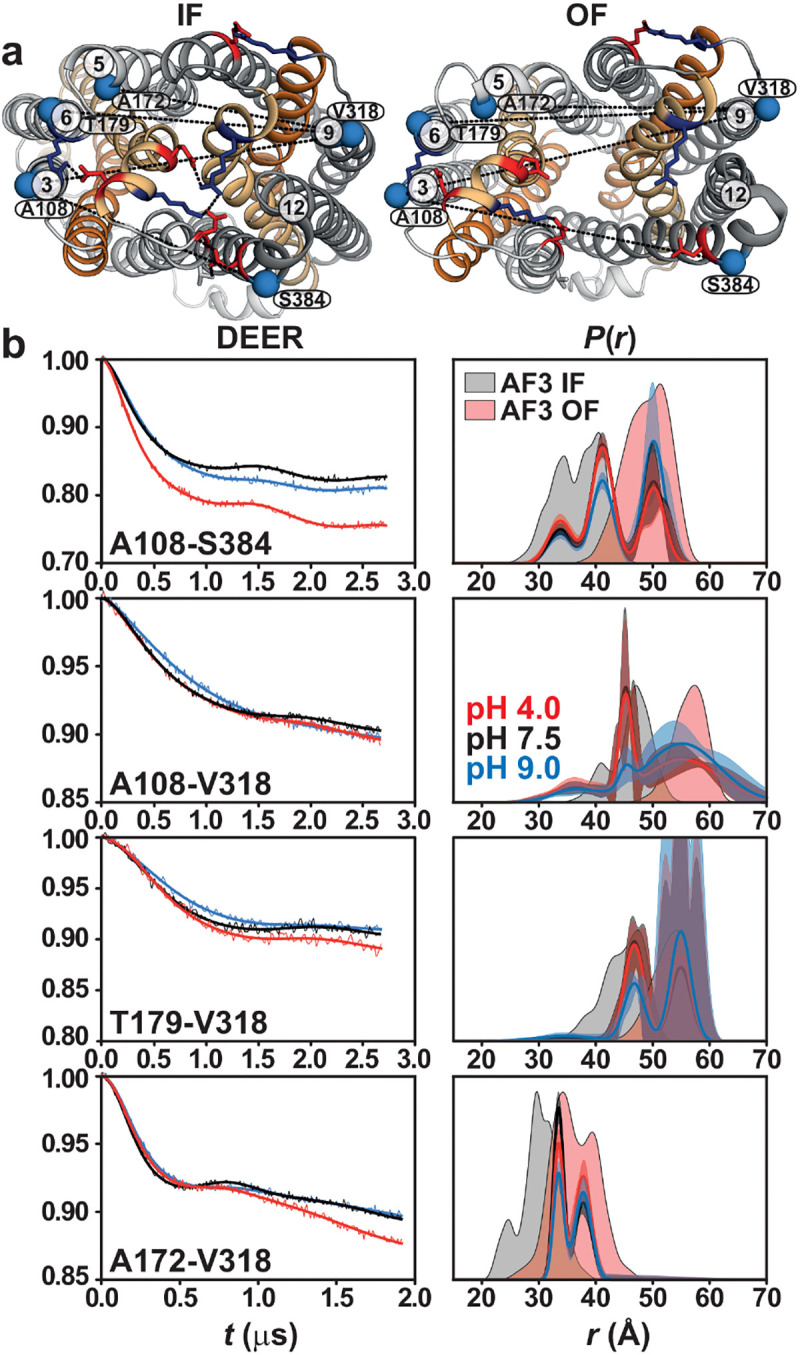
Proton binding draws the support helices closer to promote extracellular closure. (**a**) Spin-label pairs reporting on the extracellular regions of the support helices are shown as blue spheres on the IF and OF models. (**b**) Raw DEER decays with fits (left) and corresponding distance distributions, *P*(*r*) (right). Distance distributions predicted from the IF and OF models are shaded gray and red, respectively.

**Figure 8.**
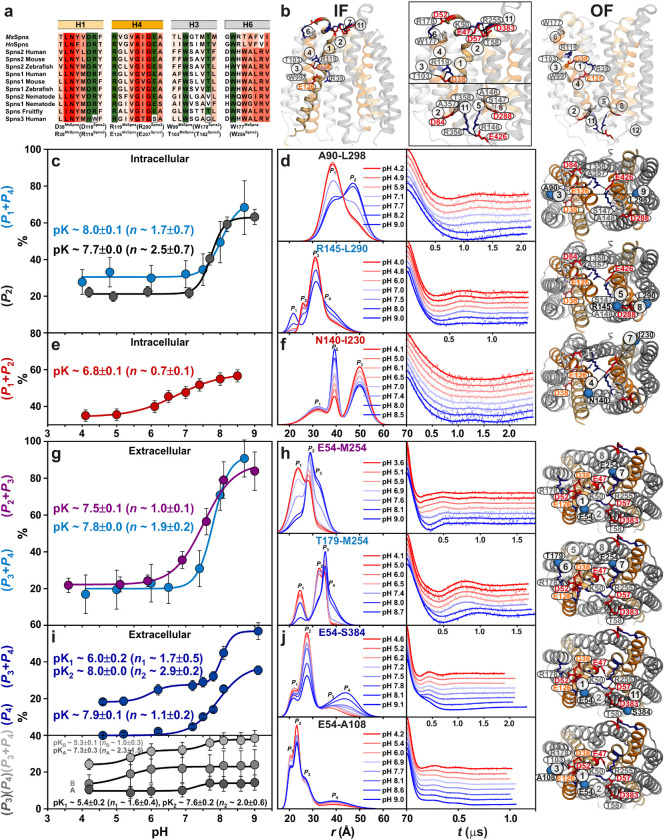
Distinct proton sensitivity and cooperativity within the *Ms*Spns substrate-binding cavity. (**a**) Sequence alignment highlighting conserved residues of the membrane-embedded protonation switches and the putative periplasmic proton-translocation pathway, shaded in green. (**b**) Membrane views of the IF and OF models showing the membrane-embedded protonation switches and proton-sensing networks on both sides of the membrane. (**c**,**d**) From right to left, baseline-corrected and normalized DEER traces with fits, corresponding distance distributions, and pH-dependent population changes for the intracellular support-helix pair A90–L298 and gating pair R145–L290. Changes in the population of the rising distance peaks were used to estimate the pK values of conformational transitions on either side of the membrane. (**e**,**f**) Intracellular substrate-binding cavity pair N140–1230. (**g**,**h**) Extracellular substrate-binding cavity pair E54–M254, which directly reports on the conserved D57:R255 salt bridge that maintains extracellular closure, and extracellular pair T179–M254, which indirectly reports on the TM5/8 lateral gate. (**i**,**j**) Extracellular TM2/11 gating pair E54–S384 and TM1/3 pair E54–A108, which probes opening of the putative periplasmic proton-translocation pathway along TMs 1, 3, and 6. Error bars in panels **c**, **e**, **g**, and **i** represent 2σ (95%) confidence intervals for the fitted peak populations shown in panels **d**, **f**, **h**, and **j**.

**Figure 9.**
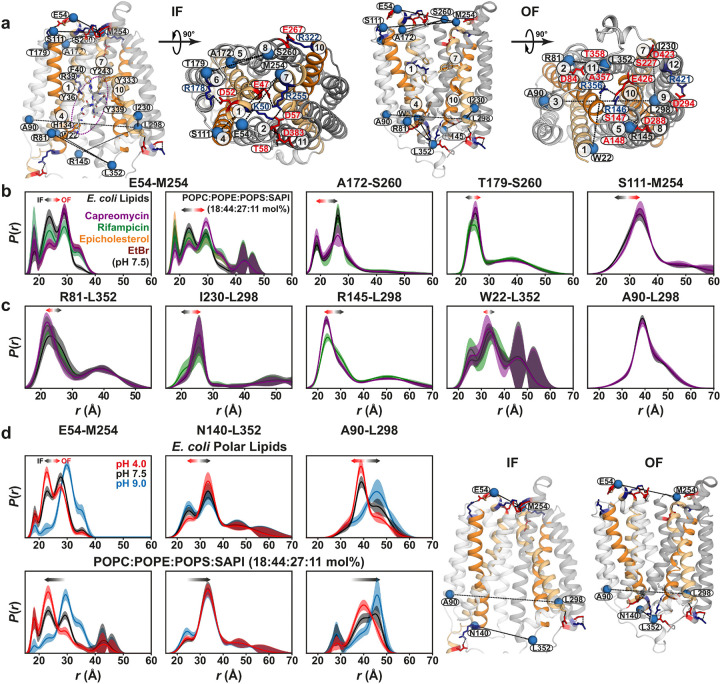
Hydrophobic and hydrophilic substrates differentially shift *Ms*Spns conformational equilibria. (**a**) Membrane and periplasmic views of the IF model and membrane and cytoplasmic views of the OF model. Capreomycin (circled) is docked in the substrate-binding cavity of the IF state, with conserved aromatic cavity residues shown as sticks. DEER spin-label pairs are shown as blue spheres. (**b**,**c**) DEER distance distributions measured at pH 7.5 in the apo state and in the presence of substrates on the extracellular (**b**) and intracellular (**c**) sides. Two lipid compositions were tested for the extracellular pair E54–M254. Water-soluble cationic substrates (capreomycin and ethidium bromide) stabilize the OF conformation, whereas lipophilic substrates (rifampicin and epicholesterol) shift the equilibrium toward the IF state. (**d**) Effects of lipid composition on *Ms*Spns conformational equilibria probed by the extracellular pair E54–M254 and the intracellular pairs N140–L352 and A90–L298. Raw DEER decays with fits are shown in Supplementary Figs. 4–6.

**Figure 10.**
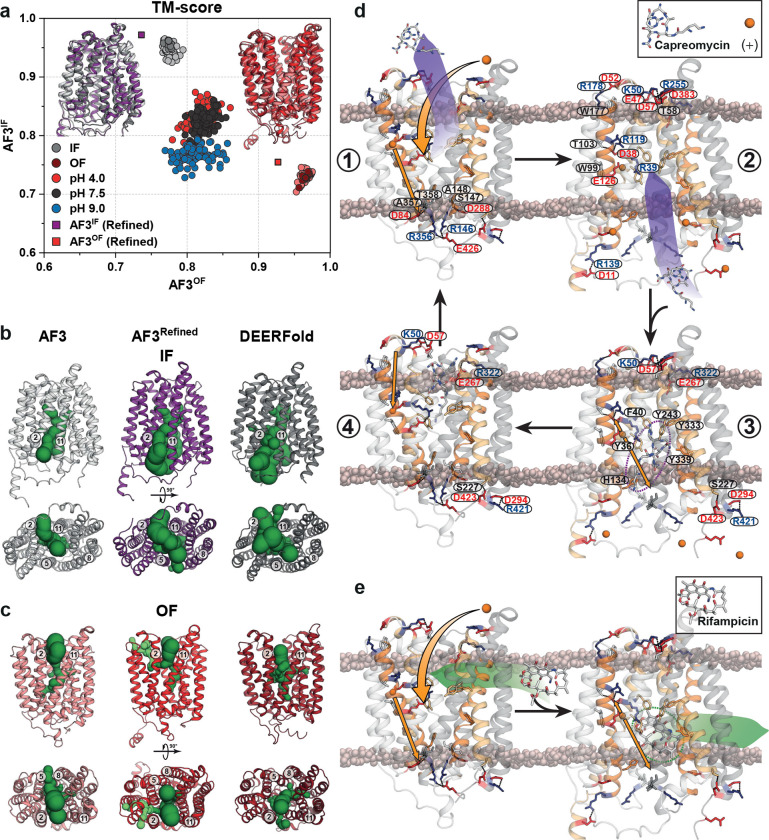
Proposed dual transport model for *Ms*Spns. (**a**) DEER-guided AlphaFold ensembles, generated by DEERFold, are compared with DEER-refined AF3 models. Models are sorted by TM-score^46^ similarity to unbiased IF and OF AF3 models, with the top 10 DEERFold IF and OF models shown in darker colors. Structural overlays of unbiased AF3 (IF: light gray, OF: light red), top DEERFold (IF: dark gray, OF: firebrick), and refined AF3 models (IF: purple, OF: red) show strong convergence. (**b**) Calculated tunnels (MOLEonline 2.5^48^, dark green) in IF models indicate that substrates can access the binding cavity from the inner membrane leaflet through the TM2/11 and TM5/8 gates or directly from the cytoplasm, allowing entry of both lipophilic and water-soluble substrates. (**c**) Tunnels calculated in OF models suggest that substrates access or exit the cavity from the outer membrane leaflet through the TM2/11 gate or directly from/to the periplasm. (**d**) Proposed antiport model for water-soluble cationic substrates. Asp38 protonation stabilizes the resting OF state, likely through a putative periplasmic proton tunnel involving conserved residues on TMs 3 and 6 (panel c, lime tunnel in AF3-refined model) (**1**). Glu126 protonation and proton transfer from Asp38 to intracellular acidic residues favor the higher-energy IF state and prime substrate binding (**2**). Binding of cationic substrates promotes Glu126 deprotonation and Asp38 reprotonation, disrupting the extracellular proton-sensing network and returning *Ms*Spn to the OF state (**3** to **4**). Four conserved intradomain salt bridges and hydrogen bonds remain largely intact during IF–OF isomerization (steps 3 and 4), consistent with limited proton- and substrate-dependent changes in intradomain DEER distances. (**e**) Proposed symport model for lipophilic substrates. Lipophilic substrates such as rifampicin bind from the outer membrane leaflet and, together with Glu126 protonation, drive OF-to-IF isomerization, promoting proton release to the cytoplasm and substrate release to the inner leaflet.

This transport model reveals previously unrecognized features of alternating access in Spns proteins and shows how a conserved set of proton-sensing interactions can be repurposed to support multiple transport outputs (Figs. 1, 2b, 5a, 8a)^7^. It also places *Ms*Spns within a broader MFS framework that includes proton-coupled sugar acid and sugar symporters such as *E. coli* DgoT and XylE^36,37^. The proposed regulatory role of Asp38 in *Ms*Spns and Asp41 in *Hn*Spns is reminiscent of Asp27 in XylE, while the reversible protonation of Asp46 and Glu133 in DgoT parallels the membrane-embedded protonation switches of Spns proteins^7,37,38^. Together with the recent finding that Spns2 exports S1P while importing glucose^14^, our observation that SAPI potentially shifts *Ms*Spns toward the IF state (Fig. 9d) supports a flexible model in which ligands and lipids tune transport directionality by biasing the same proton-coupled conformational landscape. Alongside our findings in *Hn*Spns^7^, these results suggest that dual transport may be an emerging feature of the Spns fold, whereby one ligand gradient can promote counter-transport of another and lessen the requirement for strict proton coupling under some conditions^2,14^. More broadly, ligand chemistry, particularly the pH-dependent balance between water solubility and lipophilicity in substrates such as S1P, may determine whether a ligand is exported, imported, or acts as an allosteric modulator^2,14^.

The dual behavior of *Ms*Spns, together with the expanding functional diversity of the Spns family, indicates that the same core mechanistic elements, including membrane-embedded protonation switches and extracellular proton-sensing networks, can power substrate movement in opposite directions. Applying this integrated structural-dynamics platform to additional bacterial, human, and orphan Spns transporters should define the shared and divergent principles that underlie Spns energy coupling, ligand directionality, and regulation. In this context, the ability of lipophilic compounds to favor IF states highlights a therapeutically important possibility: such modulators may either be transported by Spns proteins^15^ or trap them in inward-facing conformations^2,6^.

## Methods

No statistical methods were used to predetermine sample size. The experiments were not randomized. The investigators were not blinded to allocation during experiments and outcome assessment.

### Site-directed mutagenesis

Codon-optimized *Ms*Spns (GenScript) was cloned into pET19b vector encoding an N-terminal 10-His tag under control of an inducible T7 promoter. The three cysteine residues in *Ms*Spns were mutated (C95S, C325A, C412L) via site-directed mutagenesis with complementary oligonucleotide primers, yielding the CL protein. This construct was used as the template to introduce double-cysteine pairs. Substitution mutations were generated using a single-step PCR in which the entire template plasmid was replicated from a single mutagenic primer. *Ms*Spns mutants were sequenced using both T7 forward and reverse primers to confirm mutagenesis and the absence of aberrant changes. Mutants are identified by the native residue and primary sequence position followed by the mutant residue.

### Expression, purification, and labeling of *Ms*Spns

*Ms*Spns was expressed and purified using a similar protocol as previously published^7^. *Escherichia coli* C43 (DE3) cells (Sigma-Aldrich) were freshly transformed with pET19b vector encoding recombinant *Ms*Spns mutants. A transformant colony was used to inoculate Luria–Bertani (LB) media (Fisher Bioreagents) containing 0.1 mg/mL ampicillin (Gold Biotechnology), which was grown overnight (~15 h) at 34 °C and was subsequently used to inoculate 3–6 L of minimal medium A at a 1:50 dilution. Cultures were incubated while being shaken at 37 °C until they reached an absorbance at 600 nm (Abs_600nm_) of ~ 0.8, at which time *Ms*Spns expression was induced by the addition of 1 mM IPTG (Gold Biotechnology). The cultures were incubated overnight (~15 h) at 20 °C and then harvested by centrifugation. Cell pellets were resuspended in resuspension buffer (20 mM Tris⋅HCl, pH 8.0, 20 mM NaCl, 10 mM imidazole, and 10% [vol/vol] glycerol) at 15 mL per liter of culture, including 10 mM DTT, 1 mM EDTA, and 1 mM PMSF, and the cell suspension was lysed via sonication. Cell debris was removed by centrifugation at 9,000 × *g* for 10 min. Membranes were isolated from the supernatant by centrifugation at 180,000 × *g* for 1.5 h.

Membrane pellets were solubilized in resuspension buffer (15 mL/g membrane) containing 5 mM LMNG (Anatrace) and 0.5 mM DTT and incubated on ice with stirring for 1 h. Insoluble material was cleared by centrifugation at 180,000 × *g* for 30 min. The cleared extract was bound to 1.0 mL (bed volume) of Ni-NTA Superflow resin (Qiagen) at 4 °C for 2 h. After washing with 10 bed volumes of buffer containing 30 mM imidazole and 0.2 mM LMNG, *Ms*Spns was eluted with buffer containing 300 mM imidazole.

Double-cysteine mutants were labeled with two rounds of 20-fold molar excess 1-oxyl-2,2,5,5-tetramethylpyrroline-3-methyl methanethiosulfonate (Enzo Life Sciences) per cysteine on ice in the dark over a 4-h period, after which the sample was kept on ice at 4 °C overnight (~15 h) to yield the spin label side chain R1. Unreacted spin label was removed by size exclusion chromatography over a Superdex200 Increase 10/300 GL column (GE Healthcare) into 50 mM Tris/MES, pH 7.5, 75 mM NaCl, 0.2 mM LMNG, and 10% (vol/vol) glycerol buffer. Peak fractions of purified *Ms*Spns were combined and the final concentration was determined by A_280_ measurement (ε = 57,410 M^−1^⋅cm^−1^) for use in subsequent studies.

### Reconstitution of *Ms*Spns into nanodiscs

For all distance pairs, *E. coli* Polar Lipid Extract (Avanti Polar Lipids) was used. For a subset of distance pairs, 1-palmitoyl-2-oleoyl-sn-glycero-3-phosphocholine (POPC), 1-palmitoyl-2-oleoyl-sn-glycero-3-phosphoethanolamine (POPE), 1-palmitoyl-2-oleoyl-sn-glycero-3-phospho-L-serine (POPS) and L-α-phosphatidylinositol (Liver, Bovine, SAPI) (Avanti Polar Lipids) were combined in a 17.5:44:27.5:11 (mol/mol) ratio, dissolved in chloroform, evaporated to dryness on a rotary evaporator, and desiccated overnight under vacuum in the dark. The lipids were hydrated in 50 mM Tris/MES, pH 7.5, buffer to a final concentration of 20 mM, homogenized by freezing and thawing for 10 cycles, and stored in small aliquots at −80 °C. MSP1D1E3 was expressed and purified as previously described^39^ and dialyzed into 50 mM Tris/MES, pH 7.5, buffer. MSP1D1E3 was concentrated using a 10,000 MWCO filter concentrator and the final protein concentration was determined by A_280_ measurement (ε = 29,910 M^−1^⋅cm^−1^).

For reconstitution into nanodiscs, spin-labeled double-cysteine mutants in LMNG micelles were mixed with lipid mixture, MSP1D1E3, and sodium cholate in the following molar ratios: lipid:MSP1D1E3, 60:1; MSP1D1E3:*Ms*Spns, 10:1; and LMNG+cholate:lipid, 5:1. Reconstitution reactions were mixed at 4 °C for 1 h. Detergent was removed from the reaction by addition of 0.1 g/mL Biobeads (Bio-Rad) and incubation at 4 °C for 1 h. This was followed by another addition of 0.1 g/mL Biobeads with 1-h incubation, after which 0.2 mg/mL Biobeads were added and mixed overnight. The next day, 0.2 mg/mL Biobeads were added and mixed for 1 h. The reaction was filtered using a 0.45-μm filter to remove Biobeads. Full nanodiscs were separated from empty nanodiscs by size exclusion chromatography into 50 mM Tris/MES, pH 7.5, and 10% (vol/vol) glycerol buffer. The *Ms*Spns-containing nanodiscs were concentrated using Amicon ultra 100,000 MWCO filter concentrator, and then characterized using SDS/PAGE to verify reconstitution and estimate reconstitution efficiency. The concentration of spin-labeled mutants in nanodiscs was determined as described previously by comparing the intensity of the integrated continuous-wave electron paramagnetic resonance (CW-EPR) spectrum to that of the same mutant in detergent micelles^40^.

### Drug resistance assay

Resistance to toxic concentrations of capreomycin, conferred by *Ms*Spns WT and its mutants was carried out as previously described^7,28^. *Escherichia coli* BL21 (DE3) were transformed with empty pET19b vector, pET19b encoding *Ms*Spns WT, or mutant *Ms*Spns. A dense overnight culture from a single transformant was used to inoculate 10 mL of LB broth containing 0.1 mg/mL ampicillin to a starting Abs_600_ of 0.0375. Cultures were grown to Abs_600_ of 0.3 at 37 °C and expression of the encoded construct was induced with 1.0 μM IPTG (Gold Biotechnology). Expression was allowed to continue at 37 °C for 2 h, after which the Abs_600_ of the cultures was adjusted to 0.5. The cells were then used to inoculate (1:20 dilution, starting Abs_600_ = 0.025) a sterile 96-well microplate (Greiner) containing 50% LB broth, 0.1 mg/mL ampicillin, and 37.5 μg/mL of capreomycin. Microplates were incubated at 37 °C with shaking at 250 rpm for 2 to 8 h (6 h reported). The cell density (Abs_600_) was measured every 2 h on a SpectraMax i3 microplate reader and normalized to the 0 μg/mL drug well to obtain a relative absorbance, which accounts for growth behavior of the vector, WT, CL and other variants in the absence of drug. Experiments were performed at least in triplicate, and mean ± S.E.M. values were calculated; for *n* > 3, data were collected from multiple biological replicates. One-way ANOVA in OriginPro (OriginLab) showed that the population means of the *Ms*Spns mutants differed significantly at the 0.05 level, *F* (40, 234) = 28.81, *p* < 0.00001, whereas Tukey’s multiple-comparison test indicated that, in general, removal of the native cysteines and reintroduction of double-cysteine substitutions had little effect on the ability of *Ms*Spns to confer capreomycin resistance.

### Tryptophan fluorescence quenching

Purified *Ms*Spns in nanodisc buffer was adjusted to pH 4.0 or 9.0 using empirically determined volumes of citric acid or Tris, respectively, while maintaining equal protein concentrations across conditions. Samples were loaded into a quartz fluorescence cuvette (Starna Cells, catalog no. 16.40F-Q-10/Z15), and tryptophan fluorescence quenching was measured at 23 °C using a Horiba Scientific Fluoromax-4 spectrofluorometer. Excitation was at 295 nm, and emission was recorded from 310 to 370 nm. Fluorescence intensity at 335 nm was extracted from each spectrum to quantify the difference between pH 9.0 and pH 4.0 samples^28^. Experiments were performed in triplicate, and mean ± S.E.M. values were calculated; for *n* > 3, data were collected from multiple biological replicates. One-way ANOVA in OriginPro (OriginLab) showed that the population means of spin-labeled cysteine pairs were significantly different at the 0.05 level, *F* (40, 166) = 1160.9, *p* < 0.00001, whereas Tukey’s multiple-comparison test indicated that the constructs in nanodiscs did not differ significantly from CL *Ms*Spns.

### CW-EPR and DEER spectroscopy

CW-EPR spectra of spin-labeled *Ms*Spns samples were collected at room temperature on a Bruker EMX spectrometer operating at X-band frequency (9.5 GHz) using 10-mW incident power and a modulation amplitude of 1.6 G. DEER spectroscopy was performed on an Elexsys E580 EPR spectrometer operating at Q-band frequency (33.9 GHz) with the dead-time free four-pulse sequence at 83 K^41^. Pulse lengths were 20 ns (π/2) and 40 ns (π) for the probe pulses and 40 ns for the pump pulse. The frequency separation was 63 MHz. To ascertain the role of H^+^, samples were titrated to pH 4 and 9 with empirically determined amounts of 1 M citric acid and 1 M Tris, respectively, and confirmed by pH microelectrode (Mettler Toledo InLab Ultra-Micro-ISM) measurement. The substrate-bound state was generated by addition of 1 mM substrates at pH 7.5 or 9.0. Samples for DEER analysis were cryoprotected with 24% (vol/vol) glycerol and flash-frozen in liquid nitrogen.

Primary DEER decays were analyzed using home-written software operating in the Matlab (MathWorks) environment as previously described^42^. Briefly, the software carries out global analysis of the DEER decays obtained under different conditions for the same spin-labeled pair. The distance distribution is assumed to consist of a sum of Gaussians, the number and population of which are determined based on a statistical criterion. The generated confidence bands were determined from calculated uncertainties of the fit parameters. We also analyzed DEER decays individually and found that the resulting distributions agree with those obtained from global analysis. Comparison of the experimental distance distributions with the *Ms*Spns unbiased AF3 or DEER-guided refined models using a rotamer library approach was facilitated by the MMM software package^43^. Rotamer library calculations were conducted at 298 K.

### DEER-guided AlphaFold modeling

DEER-guided modeling of *Ms*Spns was performed using DEERFold^24^, a fine-tuned AlphaFold2-based^26^ network that incorporates experimental DEER distance distributions as pairwise distograms within the Evoformer module, together with the multiple sequence alignment (MSA), to bias structure prediction toward spin-label-constrained conformations. The DEERFold architecture, training strategy, and input-encoding scheme have been described previously^24^ and were used here without modification.

The *Ms*Spns amino acid sequence was used as input, and its MSA was generated with the ColabFold MSA pipeline^44^. For DEERFold predictions, the MSA was subsampled to an effective sequence depth of *N*_eff_ = 10^45^. For each restraint condition described below, 100 independent models were generated using distinct random seeds, producing a conformational ensemble for that condition. Models were ranked by the Earth Mover’s Distance (EMD) between the predicted and experimental distance distributions, and the highest-ranked subset, hereafter referred to as the “top models,” was retained for downstream structural analysis.

### Single-Gaussian restraints

For each spin pair, the experimental *P*(*r*) distribution measured under conditions favoring a single dominant conformation was approximated by a single Gaussian, defined by its mean distance and standard deviation. IF-state restraints were derived from distance distributions measured at pH 4.0, whereas OF-state restraints were derived from those measured at pH 9.0. Each single-Gaussian restraint set was provided to DEERFold as an independent input, generating separate IF- and OF-restrained ensembles. Because this restraint representation matches the input format on which DEERFold was fine-tuned^24^, it was expected to yield the closest agreement with the corresponding unbiased AlphaFold3 (AF3) reference models.

### Complete multi-Gaussian restraints

To determine whether the IF/OF preference was preserved with a less reduced representation of the experimental data, we also tested DEERFold using the full set of Gaussian components obtained from multi-Gaussian fitting of each DEER decay, thereby retaining the multi-peak character and breadth of the underlying *P*(*r*) distributions. Restraints were prepared independently for each DEER condition: pH 4.0, pH 7.5, and pH 9.0. Because DEERFold was fine-tuned exclusively on single-Gaussian distance distributions, these multi-Gaussian inputs fall outside the network’s training distribution and were therefore expected to generate more dispersed ensembles with reduced per-model agreement to the AF3 references. Nevertheless, this analysis provided an independent test of whether the underlying pH-dependent conformational preference was retained.

### Comparison with unbiased AF3 models

Unbiased reference models of *Ms*Spns in the IF and OF states were generated using AlphaFold3^27^, as implemented in the AlphaFold3 server. Five independent runs were performed for each state, and the highest-pLDDT model was selected as the reference conformation. DEERFold ensembles were then compared with the corresponding AF3 references using TM-score and Cα RMSD after structural superposition^46^. For each ensemble, we report the mean TM-score across the top models, together with the TM-score and Cα RMSD values for the single best-scoring model.

### DEER-guided refinement of the IF and OF conformations

The DEER distance constraints were used to refine the AF3-generated IF and OF models using a previously published approach^25^. Refinement was carried out iteratively in MODELLER^47^. In silico spin labeling was performed with the MMM software package^43^ using a rotamer-library approach. In each iteration, rotamer ensembles were first calculated at 298 K for the spin-labeled sites, and the rotamer that best matched the mean N–O midpoint position of the full ensemble was attached to the template structure provided to MODELLER. Refinement was performed using the centers of the Gaussian components corresponding to each conformational state, together with secondary-structure restraints derived from AlphaFold models. All refined models achieved a GA341 score of 1.0. After import into MMM, rotamer ensembles were recalculated (Supplementary Figs. 7 and 8), and models were ranked by the root-mean-square deviation between the experimental distance constraints and the corresponding model-derived distances across all restraints.

## Supplementary Material

Supplement 1

## Data Availability

The generated data, including those from the DEER experiments, are available in the manuscript or the supplementary materials. The DEER data and generated models have been deposited to the Zenodo repository maintained by CERN, https://doi.org/10.5281/zenodo.20467518. Other data that support the findings of this study are available from the corresponding authors upon reasonable request.
