# Supplementary material for: Proton-coupled alternating access in a versatile mycobacterial Spns drug transporter
Source: bioRxiv. 2026 Jul 6:2026.05.09.724020. Originally published 2026 May 13. Preprint. [Version 3] doi: 10.64898/2026.05.09.724020 (PMC13192786; doi:10.64898/2026.05.09.724020)

bioRxiv preprint doi: https://doi.org/10.64898/2026.05.09.724020; this version posted July 6, 2026. The copyright holder for this preprint (which was not certified by peer review) is the author/funder, who has granted bioRxiv a license to display the preprint in perpetuity. It is made available under a CC-BY 4.0 International license.

Supplementary Information

Proton-coupled alternating access in a versatile mycobacterial Spns drug transporter

Kevin L. Jagessar[1,3], Samantha Gies[1,3], Tianqi Wu[2,4], Ian Miller[1,4], Khadijeh Dastvan[1], Reza Dastvan[1,*]

[1] Department of Biochemistry and Molecular Biology, Saint Louis University School of Medicine, St. Louis, MO, USA
[2] Center for Applied AI for Protein Dynamics, Vanderbilt University, Nashville, TN, USA
[3,4] These authors contributed equally to this work.
* To whom correspondence may be addressed. Email: reza.dastvan@health.slu.edu.

This PDF file includes:

Supplementary Figs. 1-15

bioRxiv preprint doi: https://doi.org/10.64898/2026.05.09.724020; this version posted July 6, 2026. The copyright holder for this preprint (which was not certified by peer review) is the author/funder, who has granted bioRxiv a license to display the preprint in perpetuity. It is made available under a CC-BY 4.0 International license.

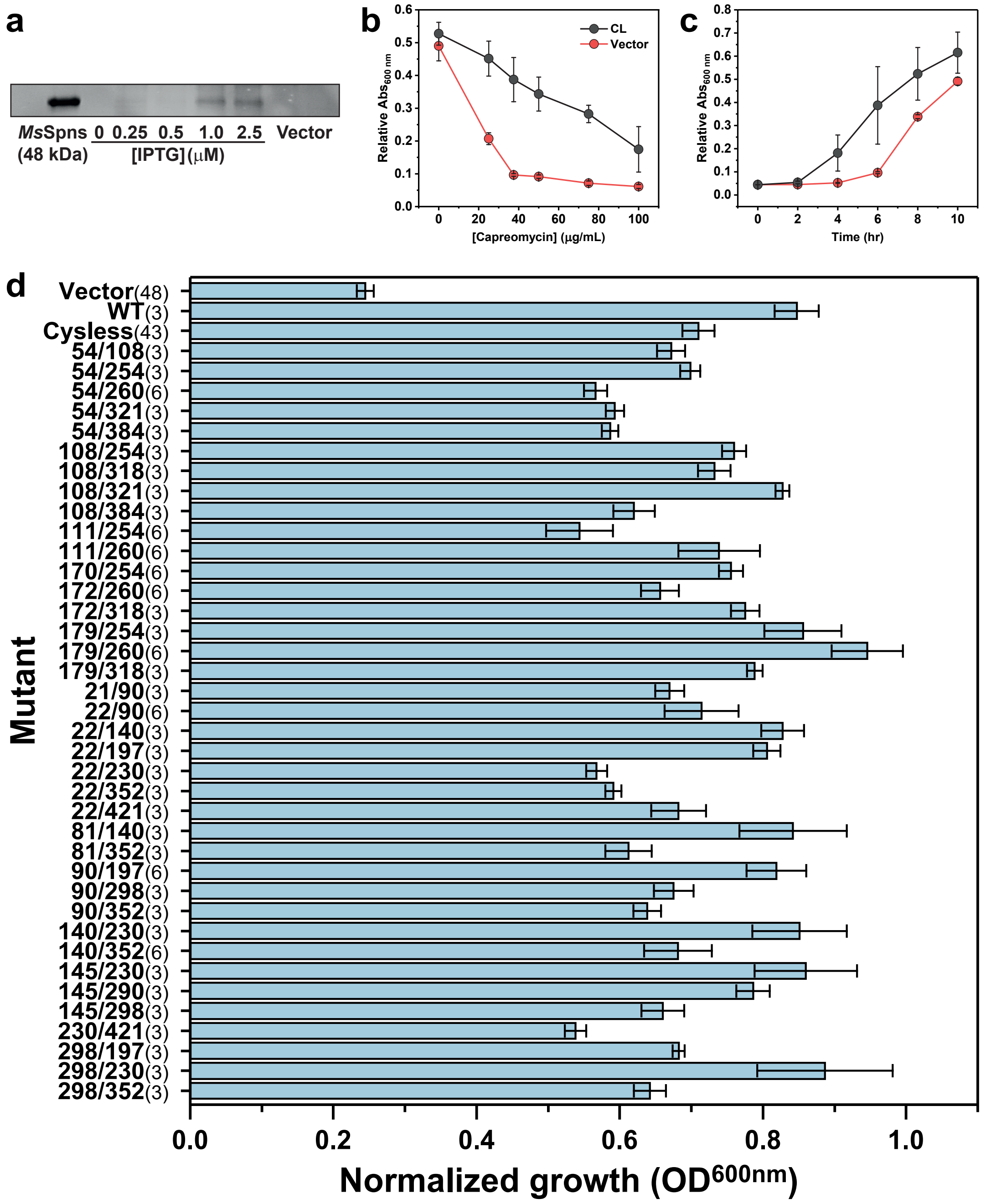

Supplementary Figure 1. Capreomycin resistance profiles of *Ms*Spns mutants used for DEER spectroscopy. (**a**) Expression of cysteine-less (CL) *Ms*Spns in cell-growth assays at different IPTG concentrations (0–2.5 μM), visualized by SDS–PAGE and InVision His-tag staining. Purified CL *Ms*Spns is shown as a standard. 1 μM IPTG was used for protein expression in growth assays. (**b**) Expression of CL *Ms*Spns enhances cell survival at elevated capreomycin concentrations relative to the vector control. Measurements were performed in triplicate after 8 h at 37 °C. A_{600nm} values were normalized to the 0 μ g/mL capreomycin control, and standard deviations are shown. The optimal capreomycin concentration was 37.5 μg/mL. (**c**) Cells expressing CL *Ms*Spns show improved growth relative to the vector control at all time points in the presence of 37.5 μg/mL capreomycin. A_{600nm} values were normalized to the 0 μg/mL control; data points represent mean ± s.d. from triplicate measurements, as in b. (**d**) Growth of *Ms*Spns mutants at 37.5 μg/mL capreomycin, normalized to the 0 μg/mL control. Bars show mean ± S.E.M. for at least three replicates, with replicate numbers indicated in parentheses. For $n > 3$, data were collected from multiple biological replicates. One-way ANOVA showed significant differences among mutants at the 0.05 level $F(40, 234) = 28.81$, $p < 0.00001$, whereas Tukey's multiple-comparison test indicated that the double-cysteine mutants did not differ significantly from WT or CL *Ms*Spns, showing that cysteine substitutions generally do not impair capreomycin resistance.

bioRxiv preprint doi: https://doi.org/10.64898/2026.05.09.724020; this version posted July 6, 2026. The copyright holder for this preprint (which was not certified by peer review) is the author/funder, who has granted bioRxiv a license to display the preprint in perpetuity. It is made available under a CC-BY 4.0 International license.

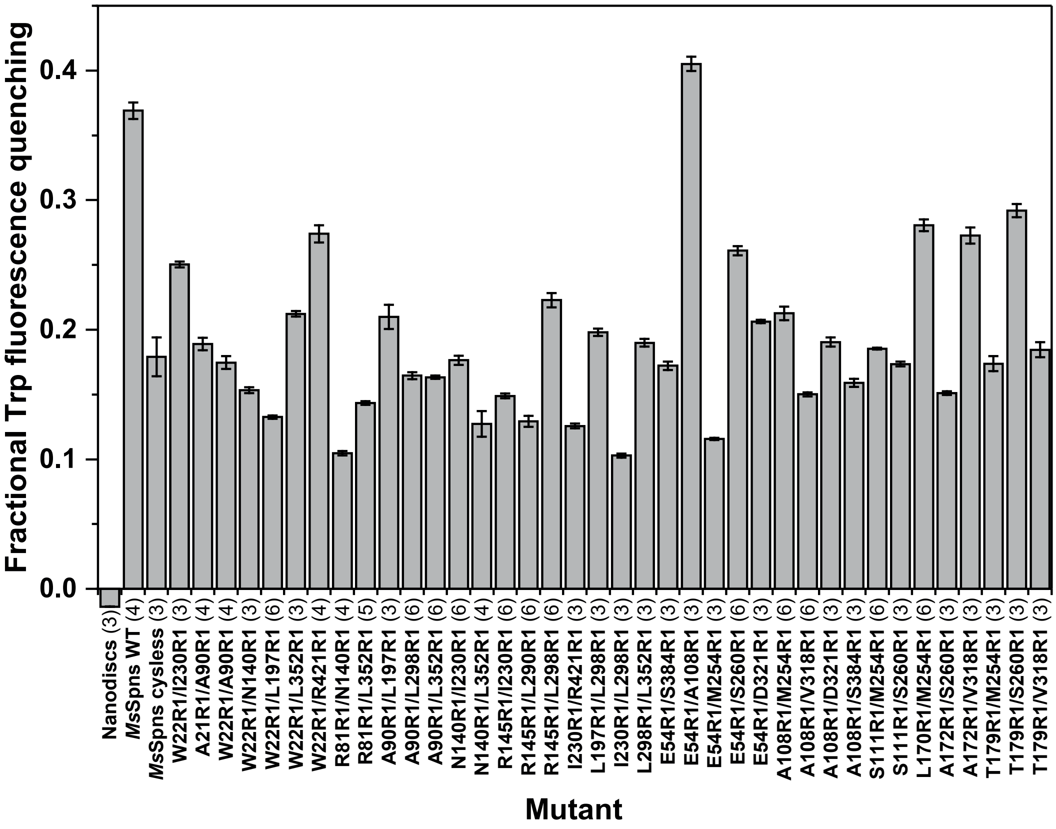

Supplementary Figure 2. Fractional fluorescence quenching of *Ms*Spns mutants. Low-pH fluorescence quenching of native tryptophan residues was used as a surrogate reporter of conformational changes in spin-labeled *Ms*Spns reconstituted in nanodiscs, as described in Methods. Bars show mean ± S.E.M. for at least three replicates, with replicate numbers indicated in parentheses. For $n > 3$, data were collected from multiple biological replicates. One-way ANOVA showed significant differences among mutants $F(40,166) = 1160.9$, $p < 0.00001$, whereas Tukey's multiple-comparison test indicated that removal of native cysteines, introduction of target-site cysteines, and spin labeling did not significantly alter conformationally coupled fluorescence quenching.

bioRxiv preprint doi: https://doi.org/10.64898/2026.05.09.724020; this version posted July 6, 2026. The copyright holder for this preprint (which was not certified by peer review) is the author/funder, who has granted bioRxiv a license to display the preprint in perpetuity. It is made available under a CC-BY 4.0 International license.

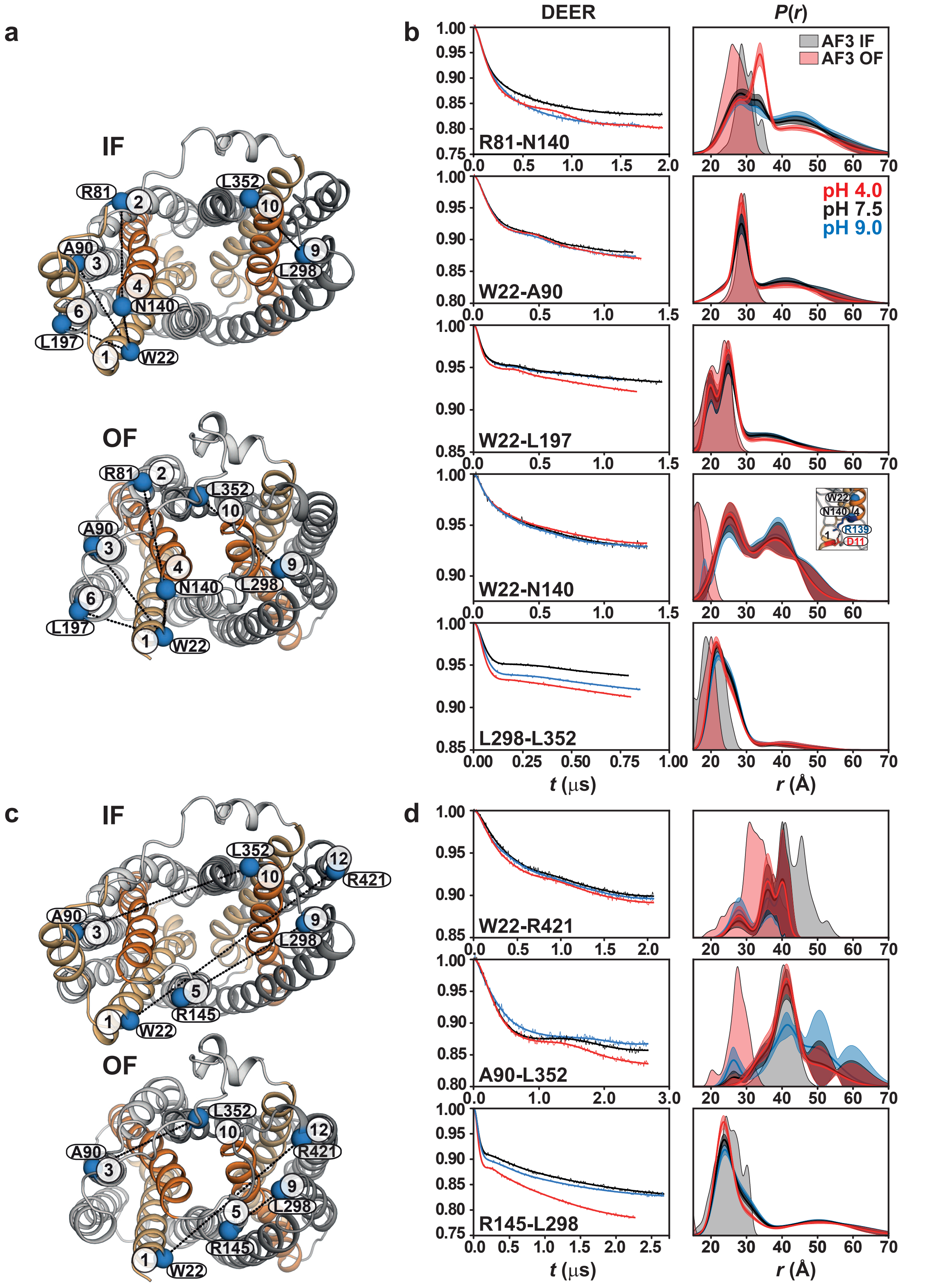

Supplementary Figure 3. Intra- and interdomain DEER distance measurements on the intracellular side. (**a**) Intradomain spin-label pairs are shown as blue spheres on the intracellular side of the IF and OF models. (**b**) Raw DEER decays with fits (left) and corresponding distance distributions, *P*(*r*), measured in lipid nanodiscs (right). Distance distributions predicted from the IF and OF models are shown as gray and red shaded regions, respectively. Most intradomain pairs show limited proton-coupled conformational changes, except for R81–L352. (**c**) Interdomain spin-label pairs are shown as blue spheres on the intracellular side of the IF and OF models. (**d**) DEER decays with fits (left) and corresponding nanodiscs distance distributions, *P*(*r*) (right), with predicted IF and OF distributions shaded gray and red, respectively.

bioRxiv preprint doi: https://doi.org/10.64898/2026.05.09.724020; this version posted July 6, 2026. The copyright holder for this preprint (which was not certified by peer review) is the author/funder, who has granted bioRxiv a license to display the preprint in perpetuity. It is made available under a CC-BY 4.0 International license.

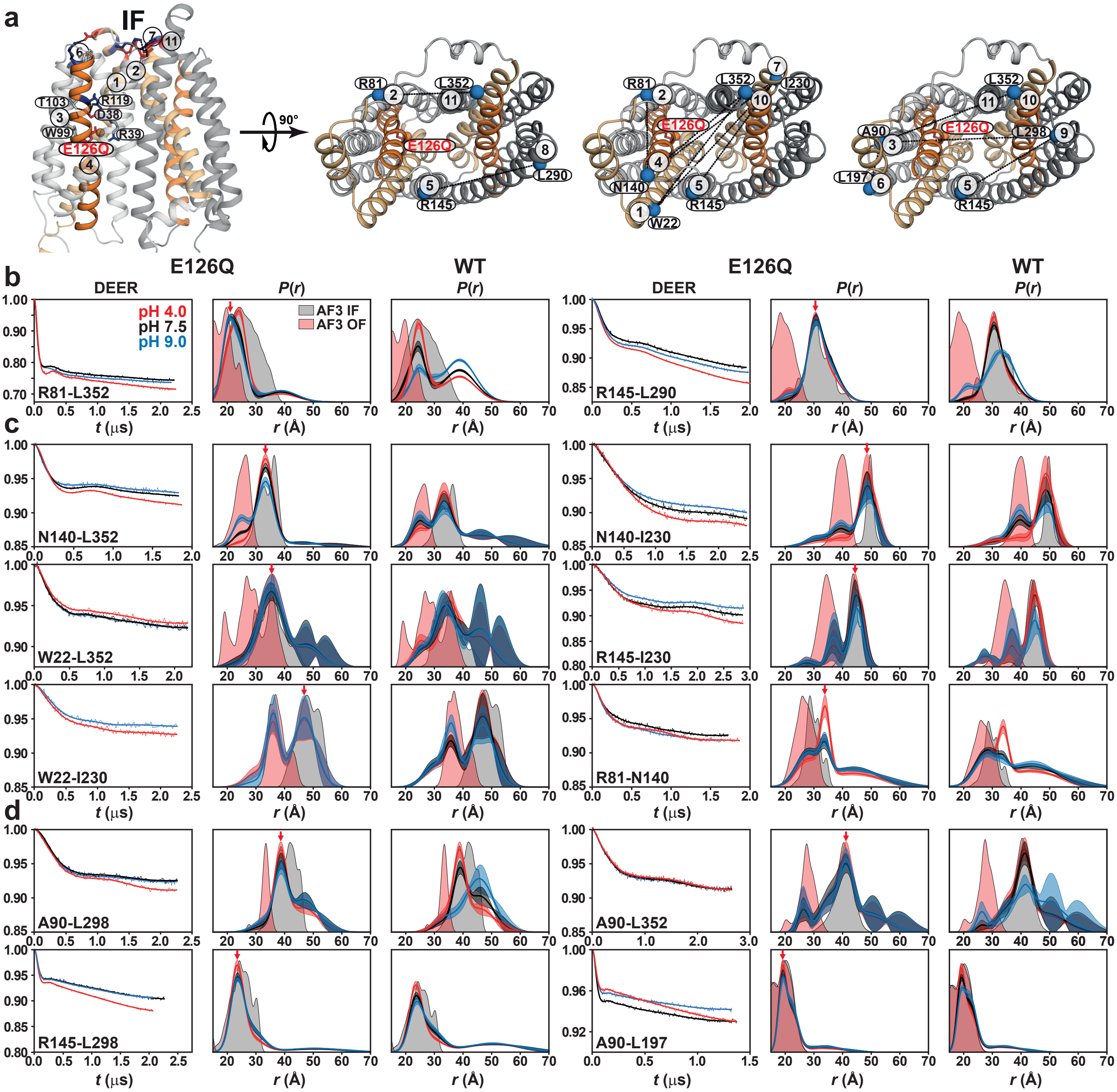

Supplementary Figure 4. Glu126 protonation opens the intracellular side of *Ms*Spns with opposing effects on intracellular gates. (**a**) Spin-label pairs reporting on intracellular conformational changes are shown as blue spheres on the IF model. (**b**–**d**) Effects of the protonation-mimetic E126Q mutation on conformational equilibria at the intracellular gates (**b**), intracellular substrate-binding cavity (**c**) and support helices (**d**). The E126Q mutation was combined with the double-cysteine mutations. DEER measurements show that Glu126 protonation stabilizes an IF conformation, supporting its role as a protonation switch. Confidence bands represent 2σ uncertainty in $P(r)$ associated with fitting of the primary DEER traces. Red arrows indicate the stabilized conformational state. Glu126 protonation closes the intracellular TM2/TM11 gate while opening the TM5/TM8 gate and expanding the substrate-binding cavity, thereby permitting substrate entry.

bioRxiv preprint doi: https://doi.org/10.64898/2026.05.09.724020; this version posted July 6, 2026. The copyright holder for this preprint (which was not certified by peer review) is the author/funder, who has granted bioRxiv a license to display the preprint in perpetuity. It is made available under a CC-BY 4.0 International license.

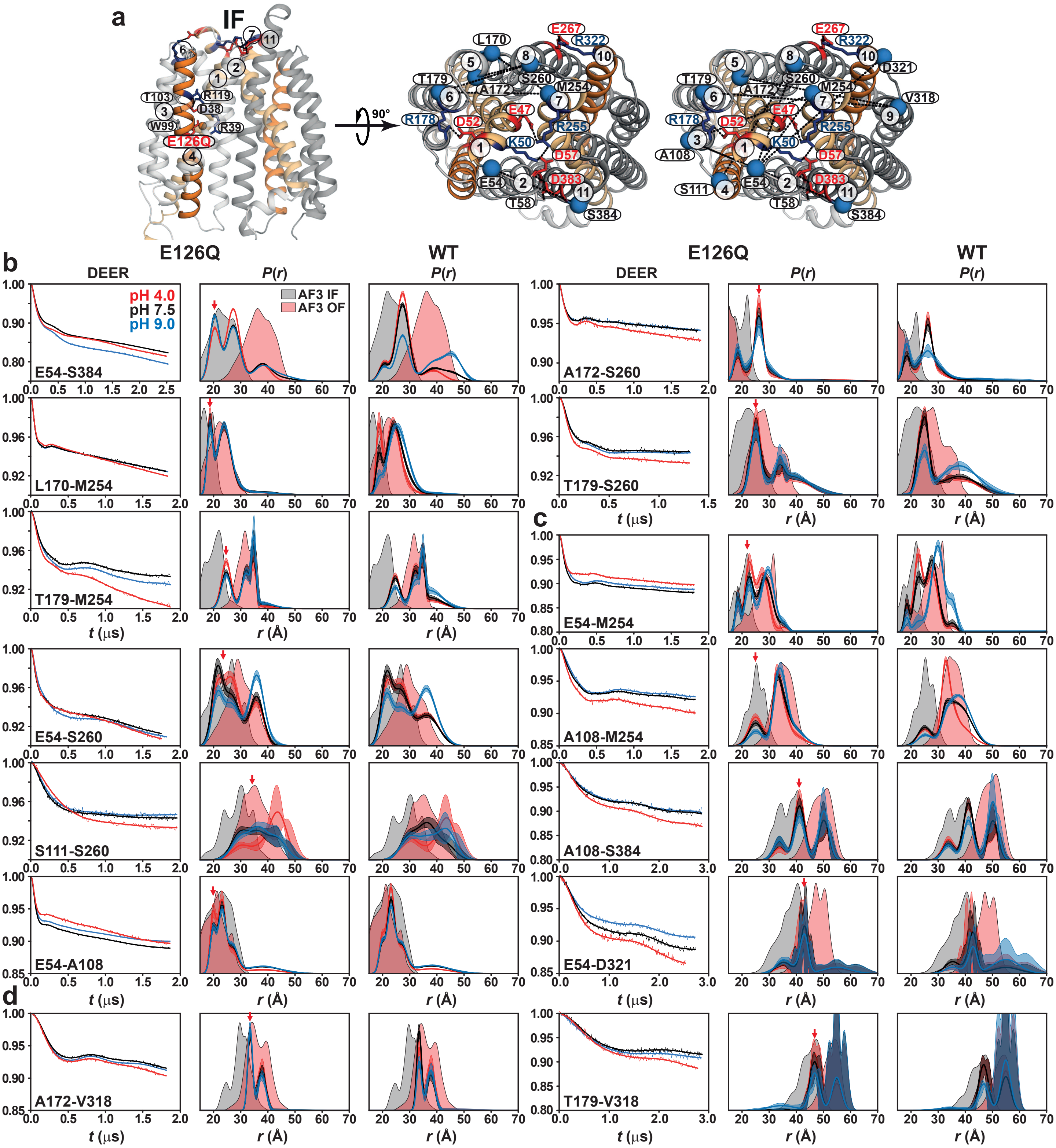

Supplementary Figure 5. Glu126 protonation promotes extracellular closure in *Ms*Spns. (**a**) Spin-label pairs reporting on extracellular conformational changes are shown as blue spheres on the IF model. (**b**–**d**) Effects of the protonation-mimetic E126Q mutation on conformational equilibria at the extracellular gates (**b**), extracellular substrate-binding cavity (**c**) and support helices (**d**). The E126Q mutation was combined with the double-cysteine mutations. DEER measurements show that Glu126 protonation stabilizes an IF conformation, supporting its role as a protonation switch. Confidence bands represent 2σ uncertainty in *P*(*r*) associated with fitting of the primary DEER traces. Red arrows indicate the stabilized conformational state.

bioRxiv preprint doi: https://doi.org/10.64898/2026.05.09.724020; this version posted July 6, 2026. The copyright holder for this preprint (which was not certified by peer review) is the author/funder, who has granted bioRxiv a license to display the preprint in perpetuity. It is made available under a CC-BY 4.0 International license.

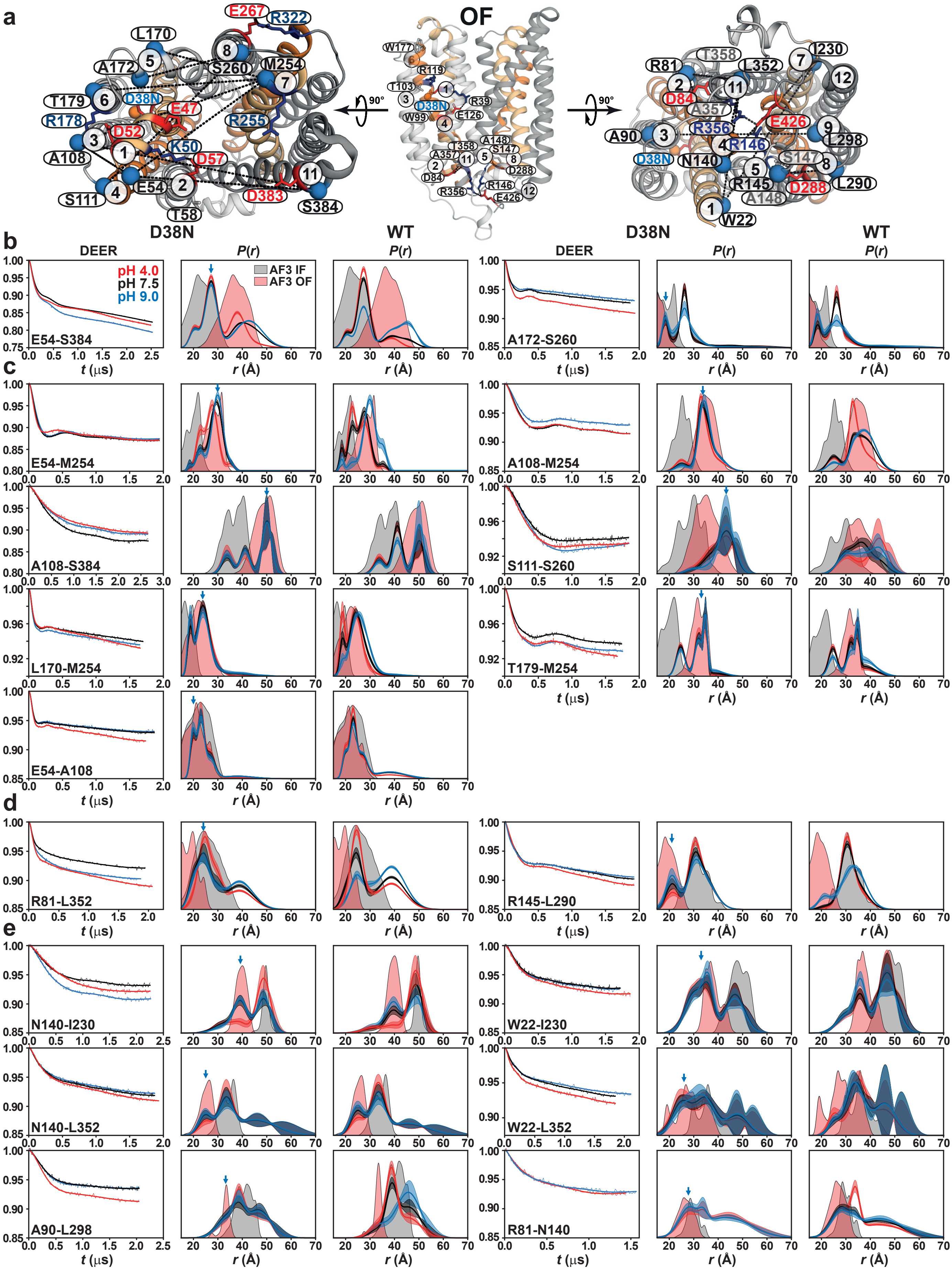

Supplementary Figure 6. Asp38 protonation stabilizes the outward-facing conformation of *Ms*Spns with opposing effects on intracellular gates. (**a**) Spin-label pairs reporting on intracellular and extracellular conformational changes. (**b**–**e**) Effects of the protonation-mimetic D38N mutation on conformational equilibria at the extracellular (**b**,**c**) and intracellular (**d**,**e**) sides. DEER measurements show that Asp38 protonation stabilizes an OF conformation, supporting its role as a protonation switch. Blue arrows indicate the stabilized conformational state. In contrast to Glu126 protonation, Asp38 protonation closes the intracellular TM5/TM8 gate and substrate-binding cavity while opening the TM2/TM11 gate.

bioRxiv preprint doi: https://doi.org/10.64898/2026.05.09.724020; this version posted July 6, 2026. The copyright holder for this preprint (which was not certified by peer review) is the author/funder, who has granted bioRxiv a license to display the preprint in perpetuity. It is made available under a CC-BY 4.0 International license.

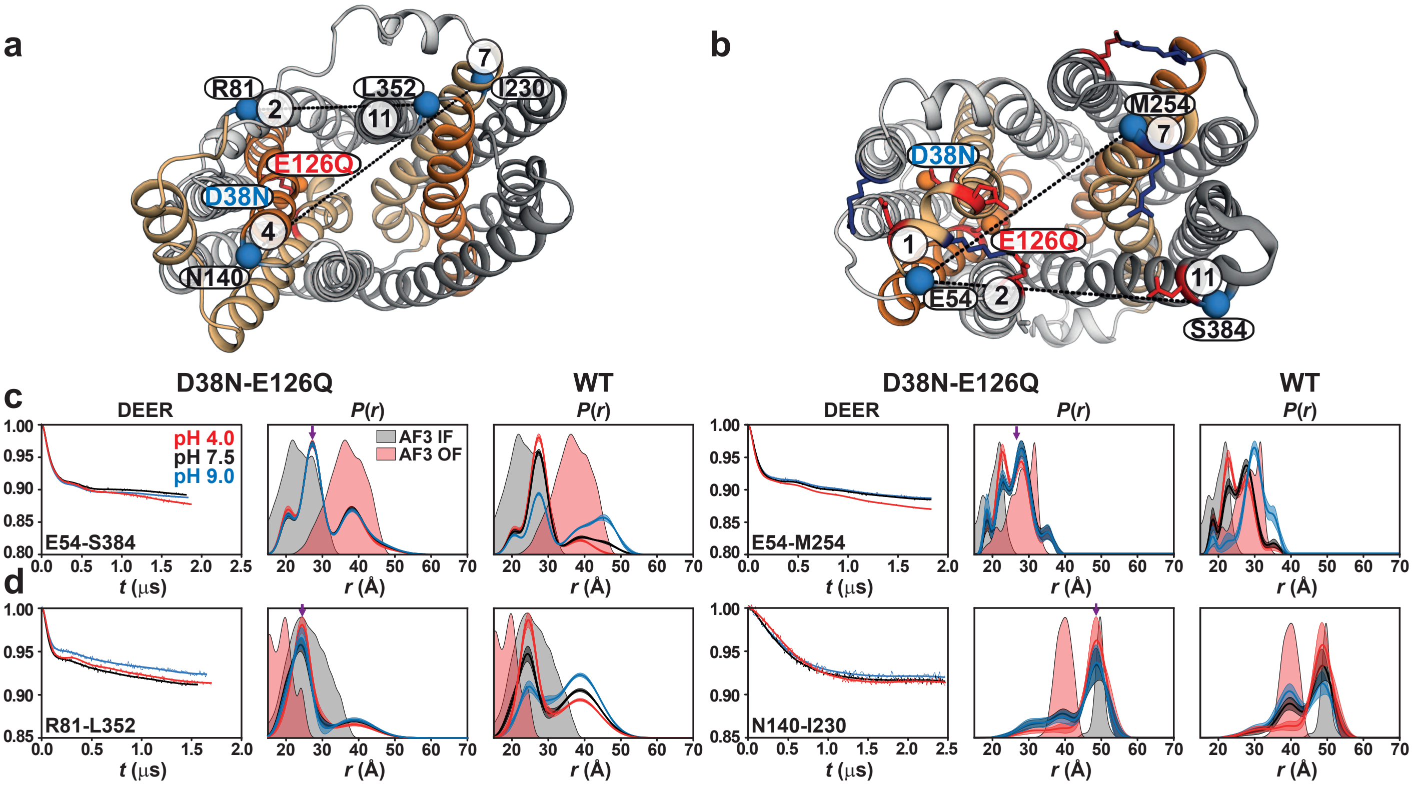

Supplementary Figure 7. Asp38 and Glu126 protonation-mimetic substitutions shift *Ms*Spns toward an IF-like intermediate. (**a**,**b**) Spin-label pairs reporting on intracellular (**a**) and extracellular (**b**) conformational changes are shown as blue spheres on representative IF and OF *Ms*Spns models, respectively. (**c**) DEER analyses of extracellular reporters show that the D38N–E126Q double mutation shifts the TM2/TM11 gate pair E54–S384 and extracellular cavity pair E54–M254 toward shorter, more IF-like distance populations, particularly under acidic conditions, while retaining longer-distance OF-like states. (**d**) DEER analyses of intracellular reporters show a similar IF-like shift for the R81–L352 TM2/TM11 gate and N140–I230 cavity pairs. Together, these data indicate that combined Asp38 and Glu126 protonation does not lock *Ms*Spns into a single canonical IF or OF state, but instead biases the ensemble toward an IF-like intermediate. These results are consistent with opposing, region-specific contributions of Asp38 and Glu126 to intracellular and extracellular conformational equilibria. Purple arrows mark mutation-enriched distance populations.

bioRxiv preprint doi: https://doi.org/10.64898/2026.05.09.724020; this version posted July 6, 2026. The copyright holder for this preprint (which was not certified by peer review) is the author/funder, who has granted bioRxiv a license to display the preprint in perpetuity. It is made available under a CC-BY 4.0 International license.

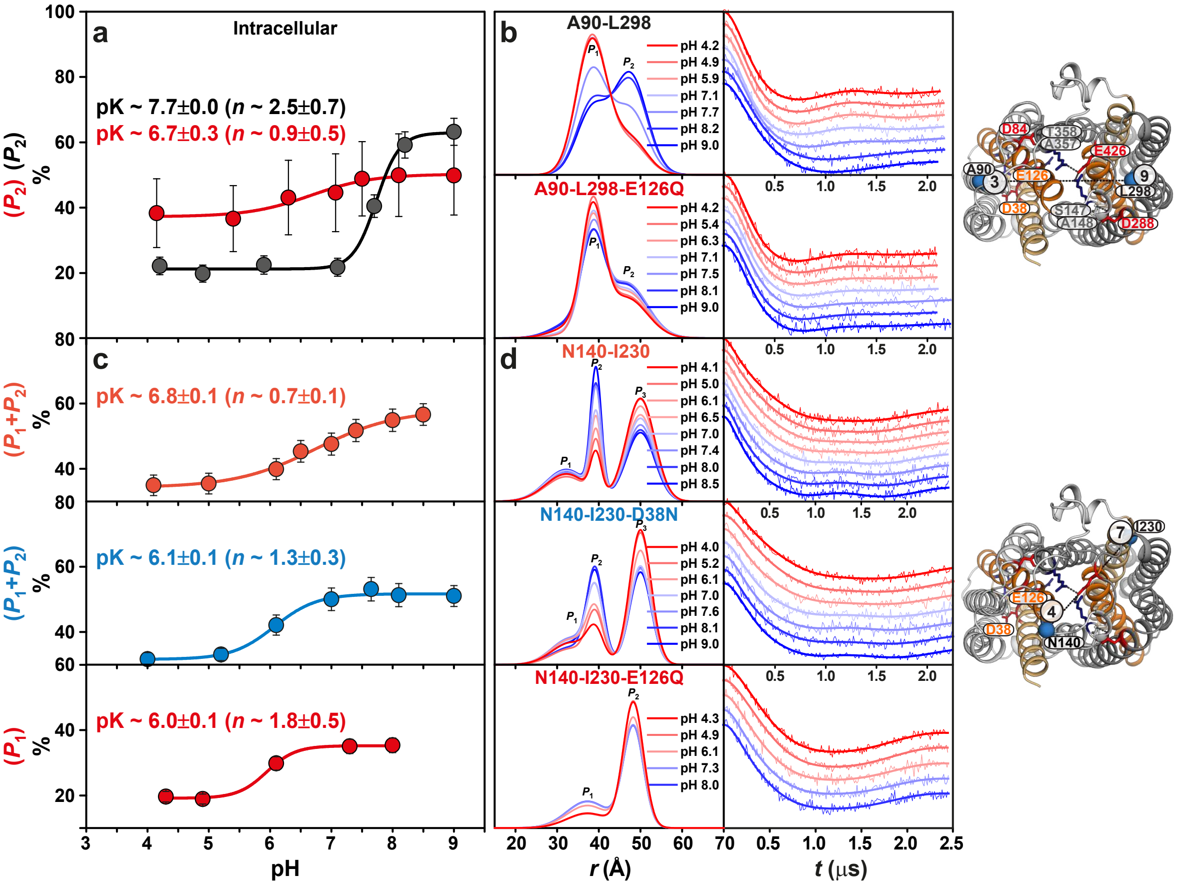

Supplementary Figure 8. Glu126 protonation regulates intracellular pH-dependent conformational changes, whereas Asp38 modestly affects the substrate-binding cavity. (**a**,**b**) Baseline-corrected and normalized DEER traces with fits, corresponding distance distributions, and pH-dependent population changes for the intracellular support-helix pair A90–L298, with and without the protonation-mimetic E126Q mutation. Population changes in the increasing-distance peaks were used to estimate apparent pK values for conformational transitions. The Glu126 protonation-mimetic mutation markedly attenuates pH-dependent rearrangement of the support helices. (**c**,**d**) Corresponding analysis of the intracellular substrate-binding cavity reporter N140–I230, with and without D38N or E126Q. The substrate-binding cavity is only modestly altered by D38N but is more strongly affected by E126Q, indicating a dominant role for Glu126 in regulating the intracellular cavity. Error bars in **a** and **c** represent 2σ (95%) confidence intervals for the fitted peak populations shown in **b** and **d**, respectively.

bioRxiv preprint doi: https://doi.org/10.64898/2026.05.09.724020; this version posted July 6, 2026. The copyright holder for this preprint (which was not certified by peer review) is the author/funder, who has granted bioRxiv a license to display the preprint in perpetuity. It is made available under a CC-BY 4.0 International license.

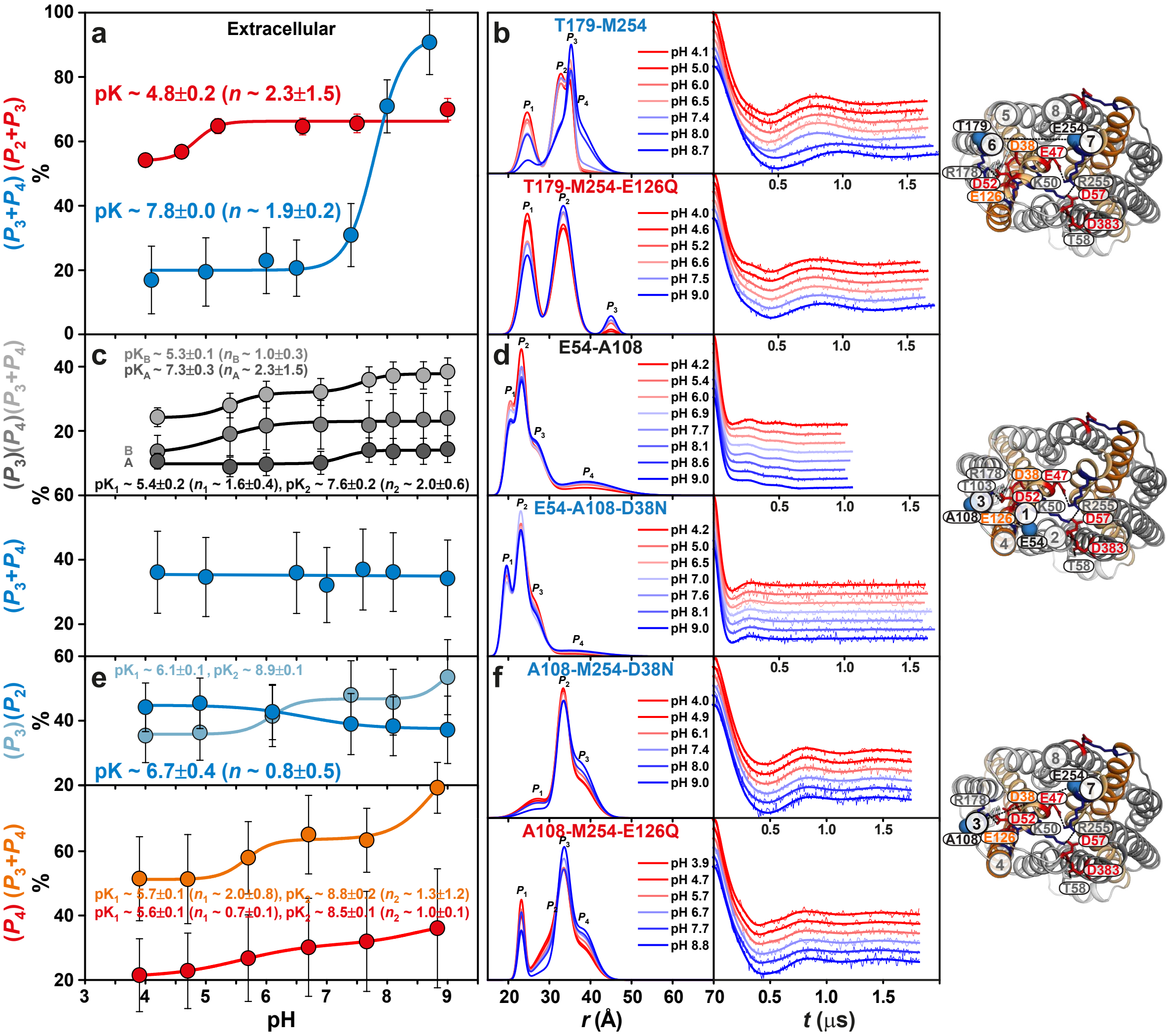

Supplementary Figure 9. Asp38 and Glu126 regulate extracellular pH-dependent conformational changes in *Ms*Spns. (**a**,**b**) Baseline-corrected and normalized DEER traces with fits, corresponding distance distributions, and pH-dependent population changes for the extracellular T179–M254 pair, which indirectly reports on the TM5/8 lateral gate, with and without the protonation-mimetic E126Q mutation. Population changes in the increasing-distance peaks were used to estimate apparent pK values for extracellular conformational transitions. As on the intracellular side, the extracellular gate and support helices are strongly modulated by Glu126 protonation. (**c**,**d**) Analysis of the extracellular TM1/3 pair E54–A108, which probes opening of the putative periplasmic proton-translocation pathway along TMs 1, 3 and 6, with and without D38N. D38N strongly suppresses pH-dependent conformational changes, supporting a dominant role for Asp38 in regulating this pathway. (**e**,**f**) Extracellular TM3/7 pair A108–M254, which reports on the extracellular substrate-binding cavity, with D38N or E126Q. D38N largely suppresses, whereas E126Q attenuates, pH-dependent conformational changes in this extracellular reporter (wildtype: Fig. 3d). Error bars in **a**, **c** and **e** represent 2σ (95%) confidence intervals for the fitted peak populations shown in **b**, **d** and **f**, respectively.

bioRxiv preprint doi: https://doi.org/10.64898/2026.05.09.724020; this version posted July 6, 2026. The copyright holder for this preprint (which was not certified by peer review) is the author/funder, who has granted bioRxiv a license to display the preprint in perpetuity. It is made available under a CC-BY 4.0 International license.

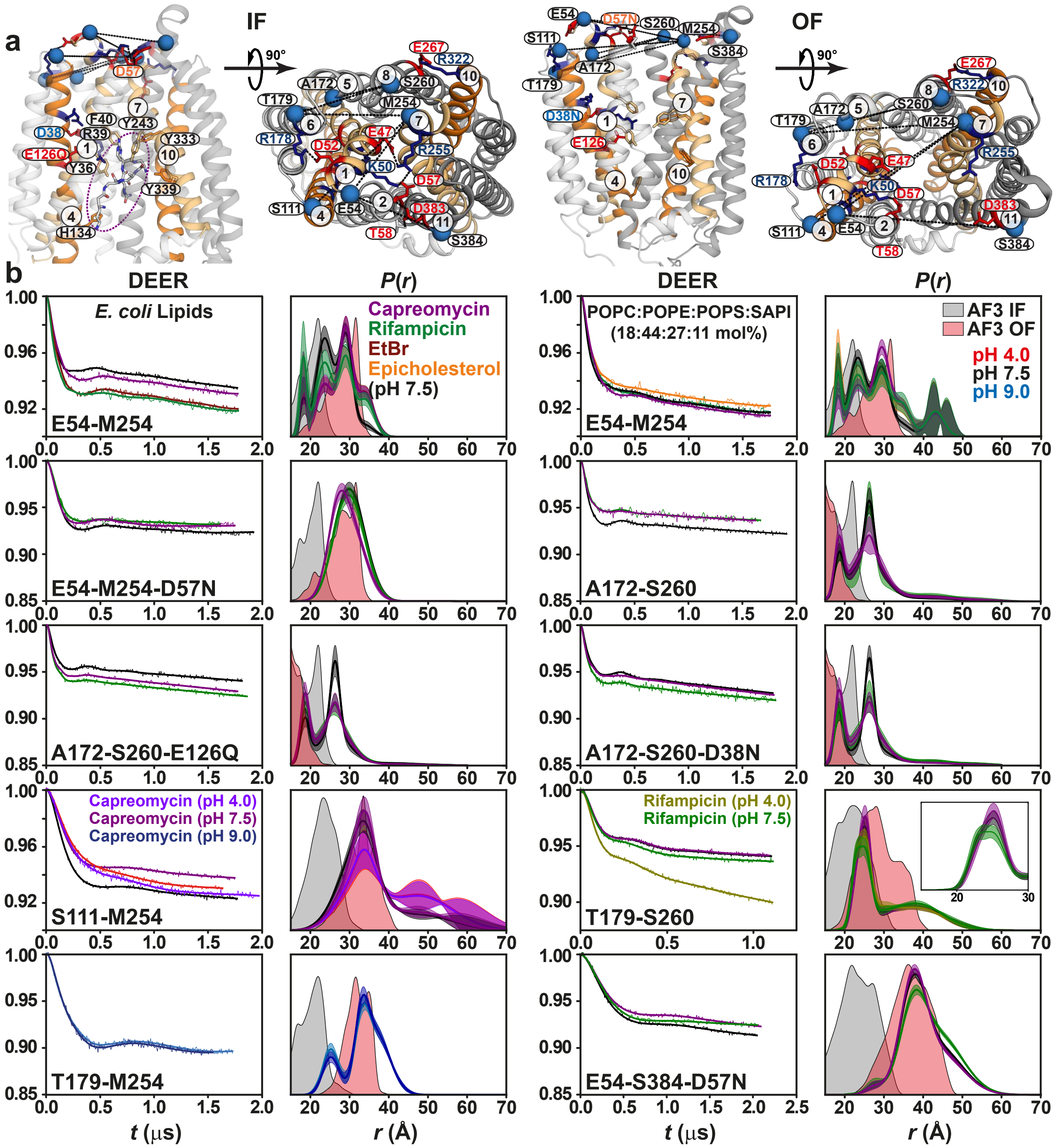

Supplementary Figure 10. Hydrophobic and hydrophilic substrates differentially shift extracellular *Ms*Spns conformational equilibria. (**a**) Membrane and periplasmic views of the IF and OF models. Capreomycin (circled) is docked in the IF substrate-binding cavity, with conserved aromatic residues in binding cavity shown as sticks and DEER spin-label pairs shown as blue spheres. Functional residue interactions stabilizing the IF conformation are highlighted in red and blue in the periplasmic views. (**b**) Raw DEER decays with fits and corresponding extracellular distance distributions measured in the apo state and in the presence of substrates, with and without protonation-mimetic mutations. Two lipid compositions were tested for the E54–M254 pair. Water-soluble cationic substrates, including capreomycin and ethidium bromide, stabilize the OF conformation, whereas lipophilic substrates, including rifampicin and epicholesterol, shift the equilibrium toward the IF state.

bioRxiv preprint doi: https://doi.org/10.64898/2026.05.09.724020; this version posted July 6, 2026. The copyright holder for this preprint (which was not certified by peer review) is the author/funder, who has granted bioRxiv a license to display the preprint in perpetuity. It is made available under a CC-BY 4.0 International license.

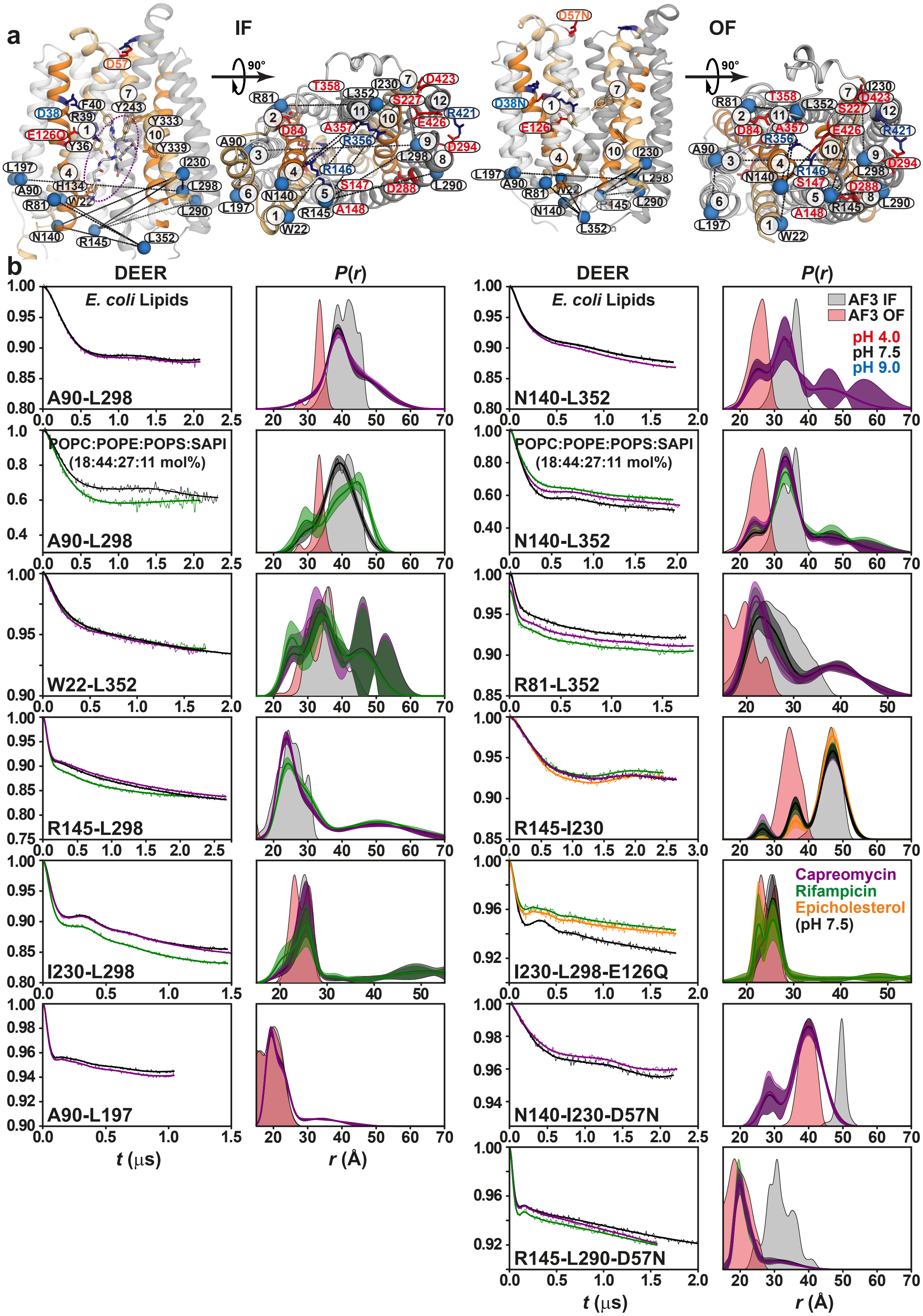

Supplementary Figure 11. Substrate hydrophobicity drives opposing shifts in intracellular *Ms*Spns conformational equilibria. (**a**) Membrane and cytoplasmic views of the IF and OF models. Capreomycin is docked in the IF substrate-binding cavity, with conserved aromatic residues shown as sticks and DEER spin-label pairs shown as blue spheres. Functional residue interactions that stabilize the OF conformation are highlighted in red and blue in the cytoplasmic views. (**b**) Raw DEER decays with fits and corresponding intracellular distance distributions measured in the apo state and in the presence of substrates, with and without protonation-mimetic mutations. Two lipid compositions were tested for the A90–L298 and N140–L352 pairs. Water-soluble cationic substrates, including capreomycin and ethidium bromide, s tabilize the OF conformation, whereas lipophilic substrates, including rifampicin and epicholesterol, shift the equilibrium toward the IF state.

bioRxiv preprint doi: https://doi.org/10.64898/2026.05.09.724020; this version posted July 6, 2026. The copyright holder for this preprint (which was not certified by peer review) is the author/funder, who has granted bioRxiv a license to display the preprint in perpetuity. It is made available under a CC-BY 4.0 International license.

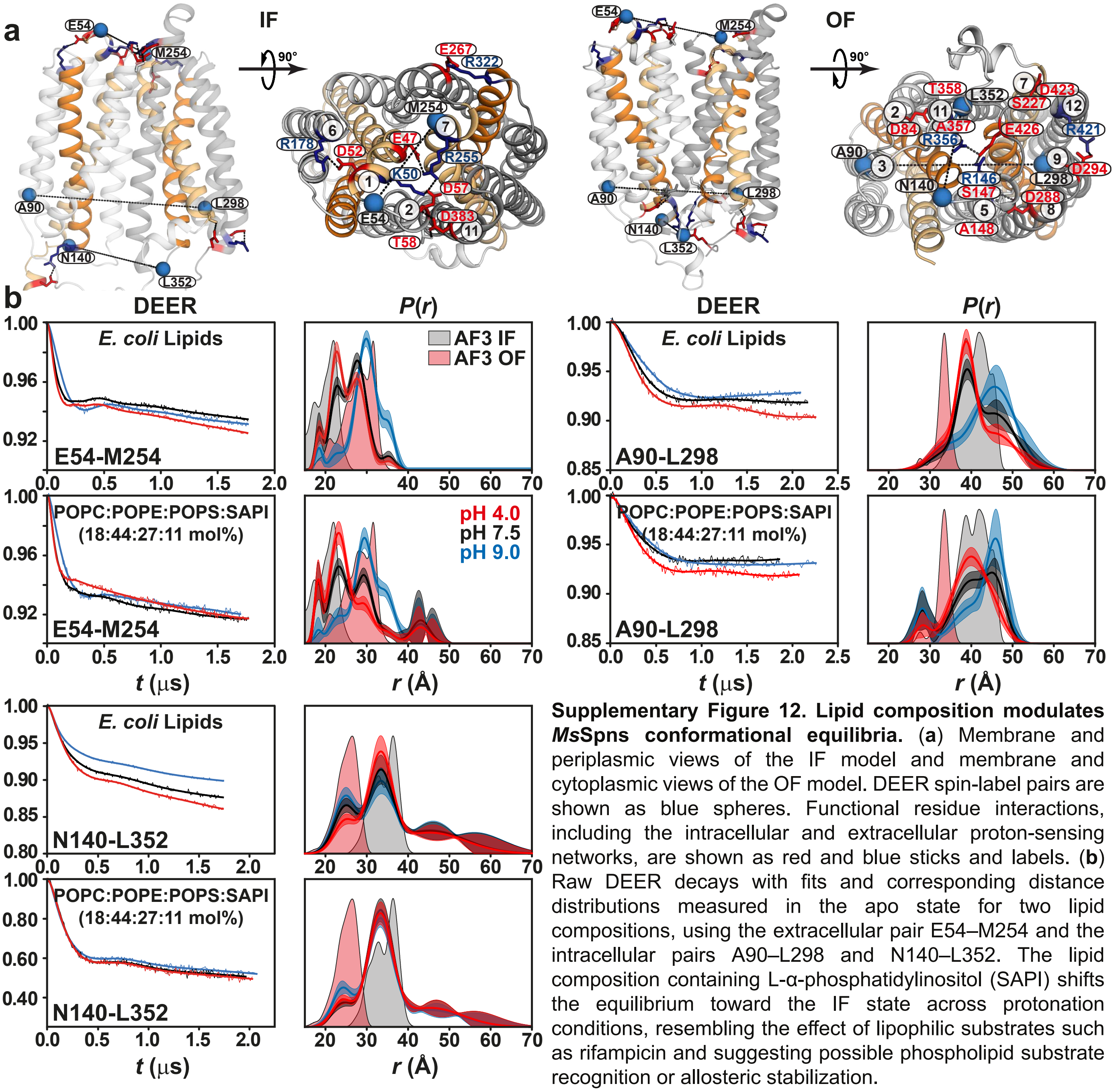

Supplementary Figure 12. Lipid composition modulates *Ms*Spns conformational equilibria. (**a**) Membrane and periplasmic views of the IF model and membrane and cytoplasmic views of the OF model. DEER spin-label pairs are shown as blue spheres. Functional residue interactions, including the intracellular and extracellular proton-sensing networks, are shown as red and blue sticks and labels. (**b**) Raw DEER decays with fits and corresponding distance distributions measured in the apo state for two lipid compositions, using the extracellular pair E54–M254 and the intracellular pairs A90–L298 and N140–L352. The lipid composition containing L-α-phosphatidylinositol (SAPI) shifts the equilibrium toward the IF state across protonation conditions, resembling the effect of lipophilic substrates such as rifampicin and suggesting possible phospholipid substrate recognition or allosteric stabilization.

bioRxiv preprint doi: https://doi.org/10.64898/2026.05.09.724020; this version posted July 6, 2026. The copyright holder for this preprint (which was not certified by peer review) is the author/funder, who has granted bioRxiv a license to display the preprint in perpetuity. It is made available under a CC-BY 4.0 International license.

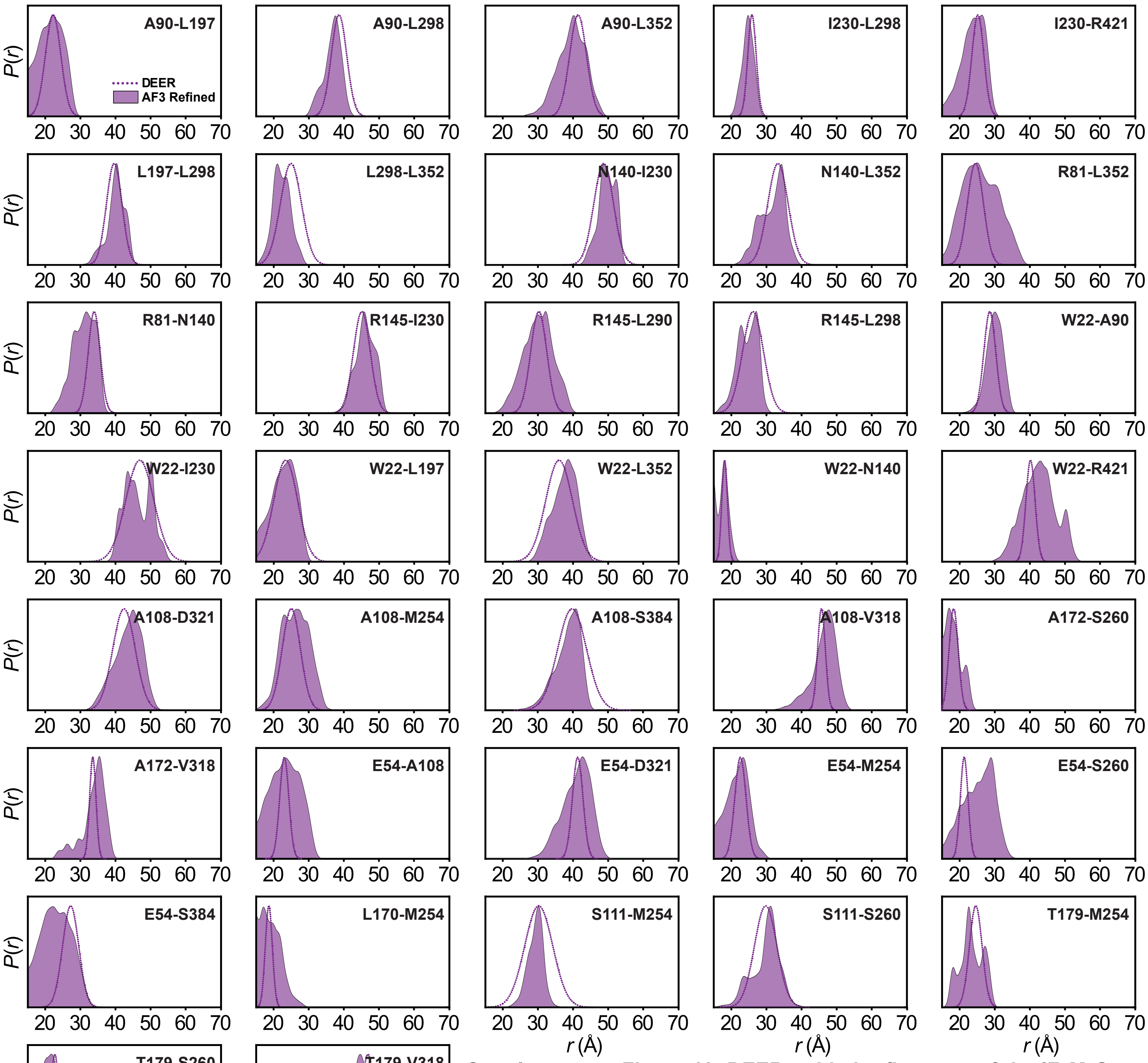

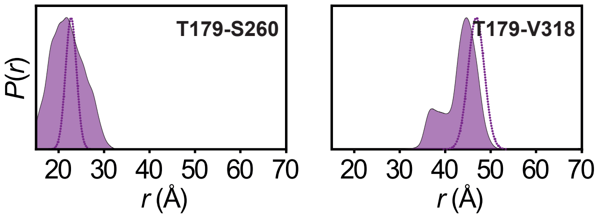

Supplementary Figure 13. DEER-guided refinement of the IF *Ms*Spns AlphaFold3 model. Distance distributions predicted from the refined IF model (purple shading) are compared with IF-associated experimental DEER distance distributions measured in nanodiscs (dotted purple lines). Overall, the refined model closely captures the experimental distance subpopulations.

bioRxiv preprint doi: https://doi.org/10.64898/2026.05.09.724020; this version posted July 6, 2026. The copyright holder for this preprint (which was not certified by peer review) is the author/funder, who has granted bioRxiv a license to display the preprint in perpetuity. It is made available under a CC-BY 4.0 International license.

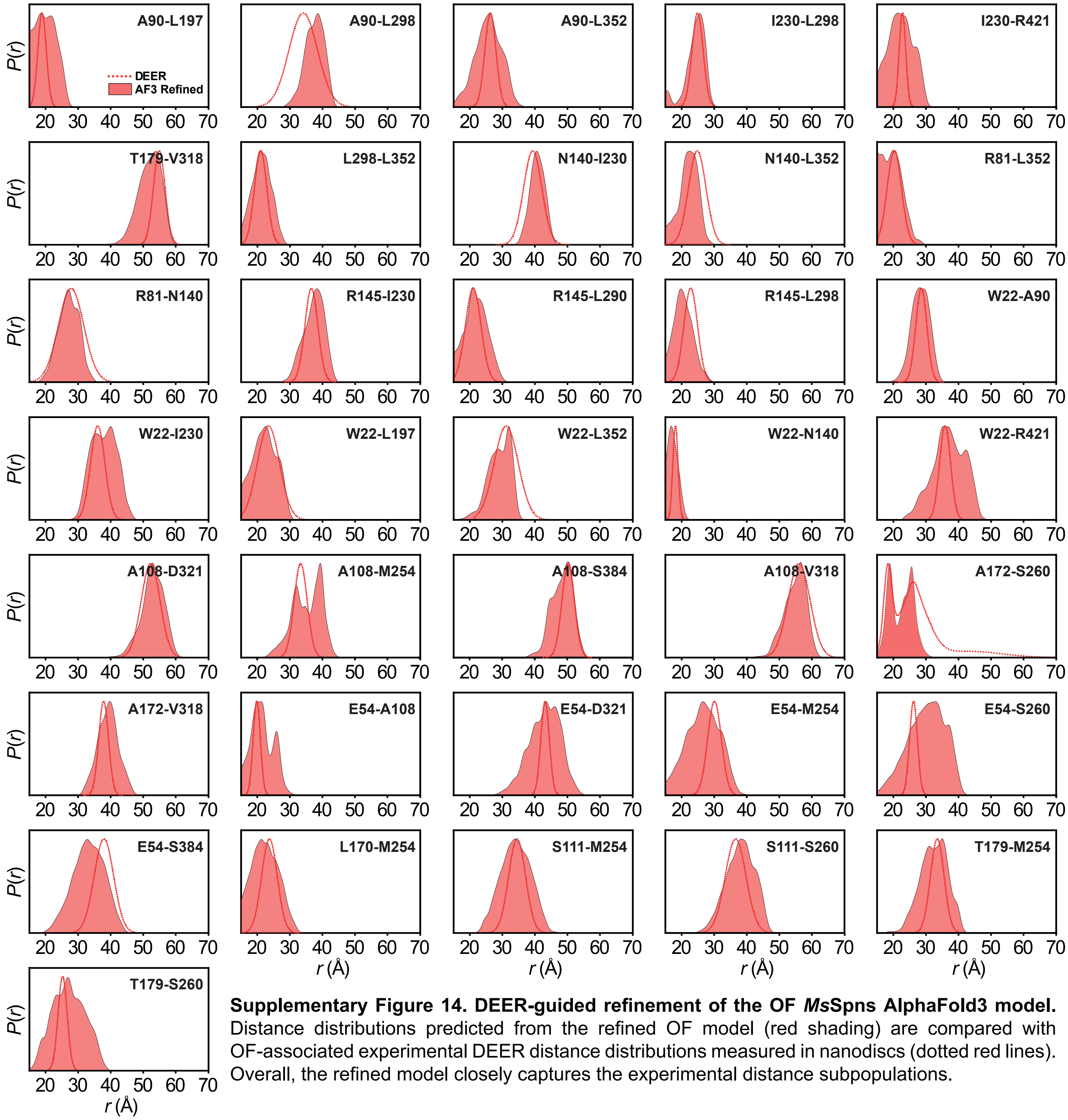

Supplementary Figure 14. DEER-guided refinement of the OF *Ms*Spns AlphaFold3 model. Distance distributions predicted from the refined OF model (red shading) are compared with OF-associated experimental DEER distance distributions measured in nanodiscs (dotted red lines). Overall, the refined model closely captures the experimental distance subpopulations.

bioRxiv preprint doi: https://doi.org/10.64898/2026.05.09.724020; this version posted July 6, 2026. The copyright holder for this preprint (which was not certified by peer review) is the author/funder, who has granted bioRxiv a license to display the preprint in perpetuity. It is made available under a CC-BY 4.0 International license.

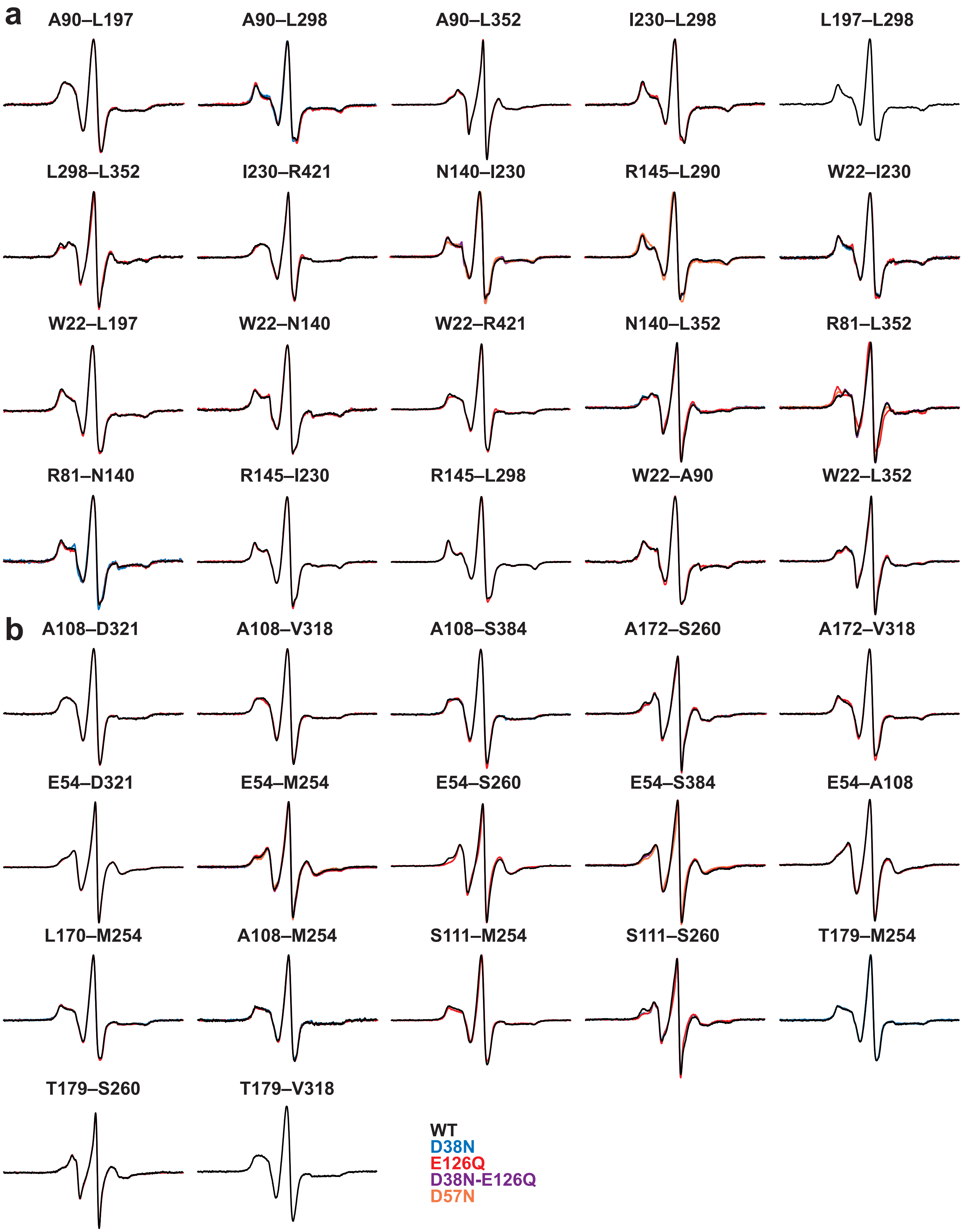

Supplementary Figure 15. CW EPR spectra of spin-labeled double-cysteine mutants for DEER spectroscopy.

bioRxiv preprint doi: https://doi.org/10.64898/2026.05.09.724020; this version posted July 6, 2026. The copyright holder for this preprint (which was not certified by peer review) is the author/funder, who has granted bioRxiv a license to display the preprint in perpetuity. It is made available under a CC-BY 4.0 International license.

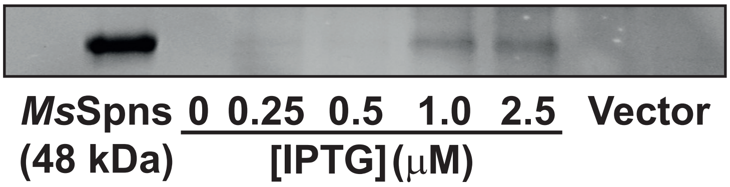

Supplementary Figure 1a

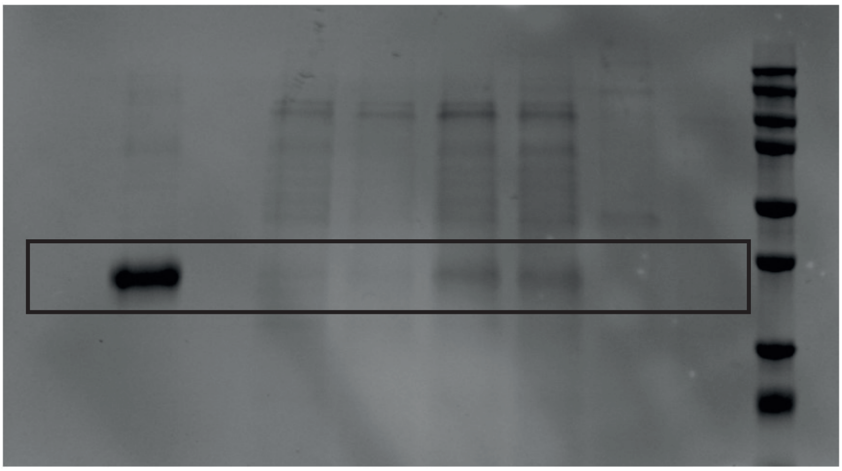

Supplement: 1 [file NIHPP2026.05.09.724020V3-supplement-1.pdf]
