# Supplementary material for: Proton-coupled alternating access in a versatile mycobacterial Spns drug transporter
Source: bioRxiv. 2026 Jun 1:2026.05.09.724020. Originally published 2026 May 13. Preprint. [Version 2] doi: 10.64898/2026.05.09.724020 (PMC13192786; doi:10.64898/2026.05.09.724020)

bioRxiv preprint doi: https://doi.org/10.64898/2026.05.09.724020; this version posted June 1, 2026. The copyright holder for this preprint (which was not certified by peer review) is the author/funder, who has granted bioRxiv a license to display the preprint in perpetuity. It is made available under a CC-BY 4.0 International license.

Supplementary Information

Proton-coupled alternating access in a versatile mycobacterial Spns drug transporter

Samantha Gies[1,3], Kevin L. Jagessar[1,3], Tianqi Wu[2], Ian Miller[1], Khadijeh Dastvan[1], Reza Dastvan[1,*]

[1] Department of Biochemistry and Molecular Biology, Saint Louis University School of Medicine, St. Louis, MO, USA
[2] Center for Applied AI for Protein Dynamics, Vanderbilt University, Nashville, TN, USA
[3] These authors contributed equally to this work.
* To whom correspondence may be addressed. Email: reza.dastvan@health.slu.edu.

This PDF file includes:

Supplementary Figs. 1-9

bioRxiv preprint doi: https://doi.org/10.64898/2026.05.09.724020; this version posted June 1, 2026. The copyright holder for this preprint (which was not certified by peer review) is the author/funder, who has granted bioRxiv a license to display the preprint in perpetuity. It is made available under a CC-BY 4.0 International license.

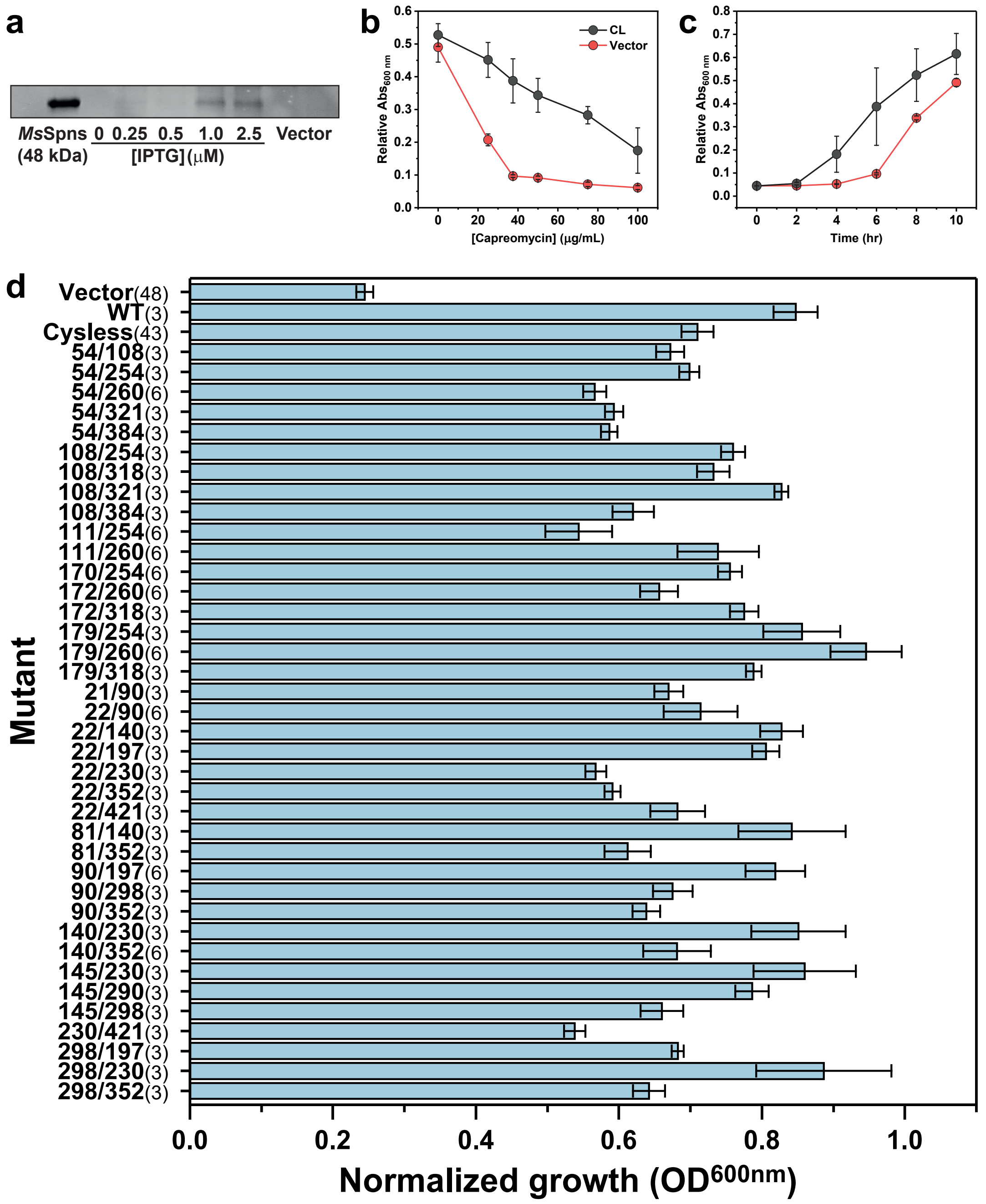

Supplementary Figure 1. Capreomycin resistance profiles of *Ms*Spns mutants used for DEER spectroscopy. (**a**) Expression of cysteine-less (CL) *Ms*Spns in cell-growth assays at different IPTG concentrations (0–2.5 μM), visualized by SDS–PAGE and InVision His-tag staining. Purified CL *Ms*Spns is shown as a standard. 1 μM IPTG was used for protein expression in growth assays. (**b**) Expression of CL *Ms*Spns enhances cell survival at elevated capreomycin concentrations relative to the vector control. Measurements were performed in triplicate after 8 h at 37 °C. A_{600nm} values were normalized to the 0 μ g/mL capreomycin control, and standard deviations are shown. The optimal capreomycin concentration was 37.5 μg/mL. (**c**) Cells expressing CL *Ms*Spns show improved growth relative to the vector control at all time points in the presence of 37.5 μg/mL capreomycin. A_{600nm} values were normalized to the 0 μg/mL control; data points represent mean ± s.d. from triplicate measurements, as in b. (**d**) Growth of *Ms*Spns mutants at 37.5 μg/mL capreomycin, normalized to the 0 μg/mL control. Bars show mean ± S.E.M. for at least three replicates, with replicate numbers indicated in parentheses. For $n > 3$, data were collected from multiple biological replicates. One-way ANOVA showed significant differences among mutants at the 0.05 level $F(40, 234) = 28.81$, $p < 0.00001$, whereas Tukey's multiple-comparison test indicated that the double-cysteine mutants did not differ significantly from WT or CL *Ms*Spns, showing that cysteine substitutions generally do not impair capreomycin resistance.

bioRxiv preprint doi: https://doi.org/10.64898/2026.05.09.724020; this version posted June 1, 2026. The copyright holder for this preprint (which was not certified by peer review) is the author/funder, who has granted bioRxiv a license to display the preprint in perpetuity. It is made available under a CC-BY 4.0 International license.

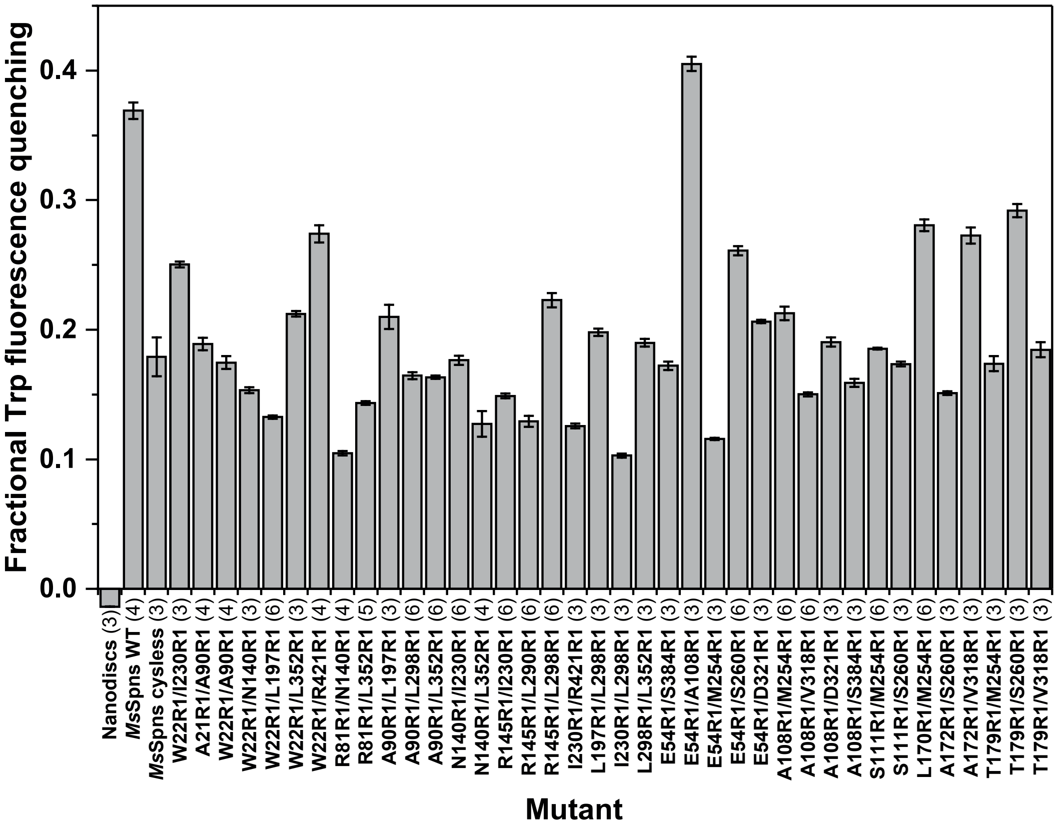

Supplementary Figure 2. Fractional fluorescence quenching of *Ms*Spns mutants. Low-pH fluorescence quenching of native tryptophan residues was used as a surrogate reporter of conformational changes in spin-labeled *Ms*Spns reconstituted in nanodiscs, as described in Methods. Bars show mean ± S.E.M. for at least three replicates, with replicate numbers indicated in parentheses. For $n > 3$, data were collected from multiple biological replicates. One-way ANOVA showed significant differences among mutants $F(40,166) = 1160.9$, $p < 0.00001$, whereas Tukey's multiple-comparison test indicated that removal of native cysteines, introduction of target-site cysteines, and spin labeling did not significantly alter conformationally coupled fluorescence quenching.

bioRxiv preprint doi: https://doi.org/10.64898/2026.05.09.724020; this version posted June 1, 2026. The copyright holder for this preprint (which was not certified by peer review) is the author/funder, who has granted bioRxiv a license to display the preprint in perpetuity. It is made available under a CC-BY 4.0 International license.

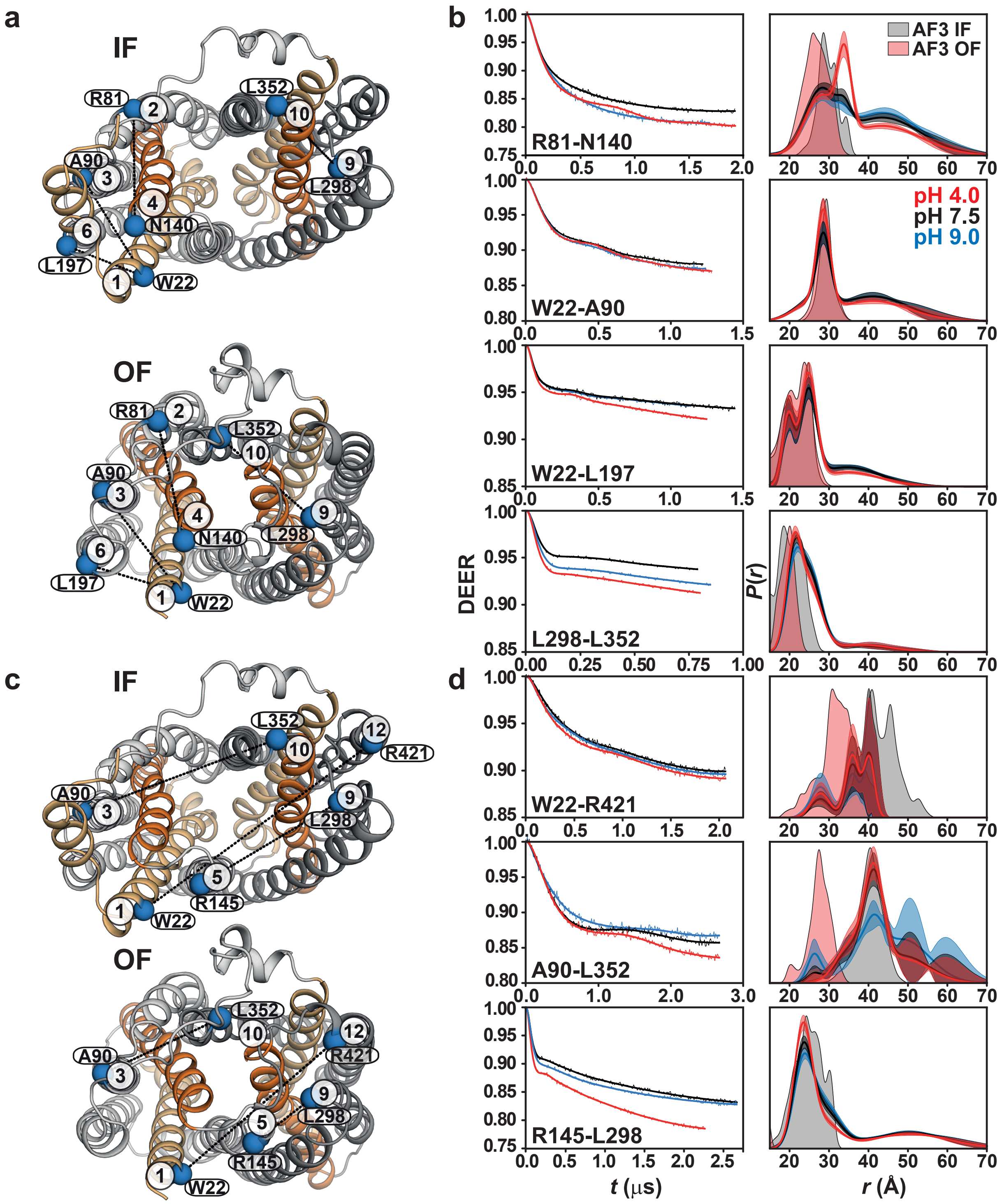

Supplementary Figure 3. Intra- and interdomain DEER distance measurements on the intracellular side. (**a**) Intradomain spin-label pairs are shown as blue spheres on the intracellular side of the IF and OF models. (**b**) Raw DEER decays with fits (left) and corresponding distance distributions, *P*(*r*), measured in lipid nanodiscs (right). Distance distributions predicted from the IF and OF models are shown as gray and red shaded regions, respectively. Most intradomain pairs show limited proton-coupled conformational changes, except for R81–L352. (**c**) Interdomain spin-label pairs are shown as blue spheres on the intracellular side of the IF and OF models. (**d**) Raw DEER decays with fits (left) and corresponding nanodiscs distance distributions, *P*(*r*) (right), with predicted IF and OF distributions shaded gray and red, respectively.

bioRxiv preprint doi: https://doi.org/10.64898/2026.05.09.724020; this version posted June 1, 2026. The copyright holder for this preprint (which was not certified by peer review) is the author/funder, who has granted bioRxiv a license to display the preprint in perpetuity. It is made available under a CC-BY 4.0 International license.

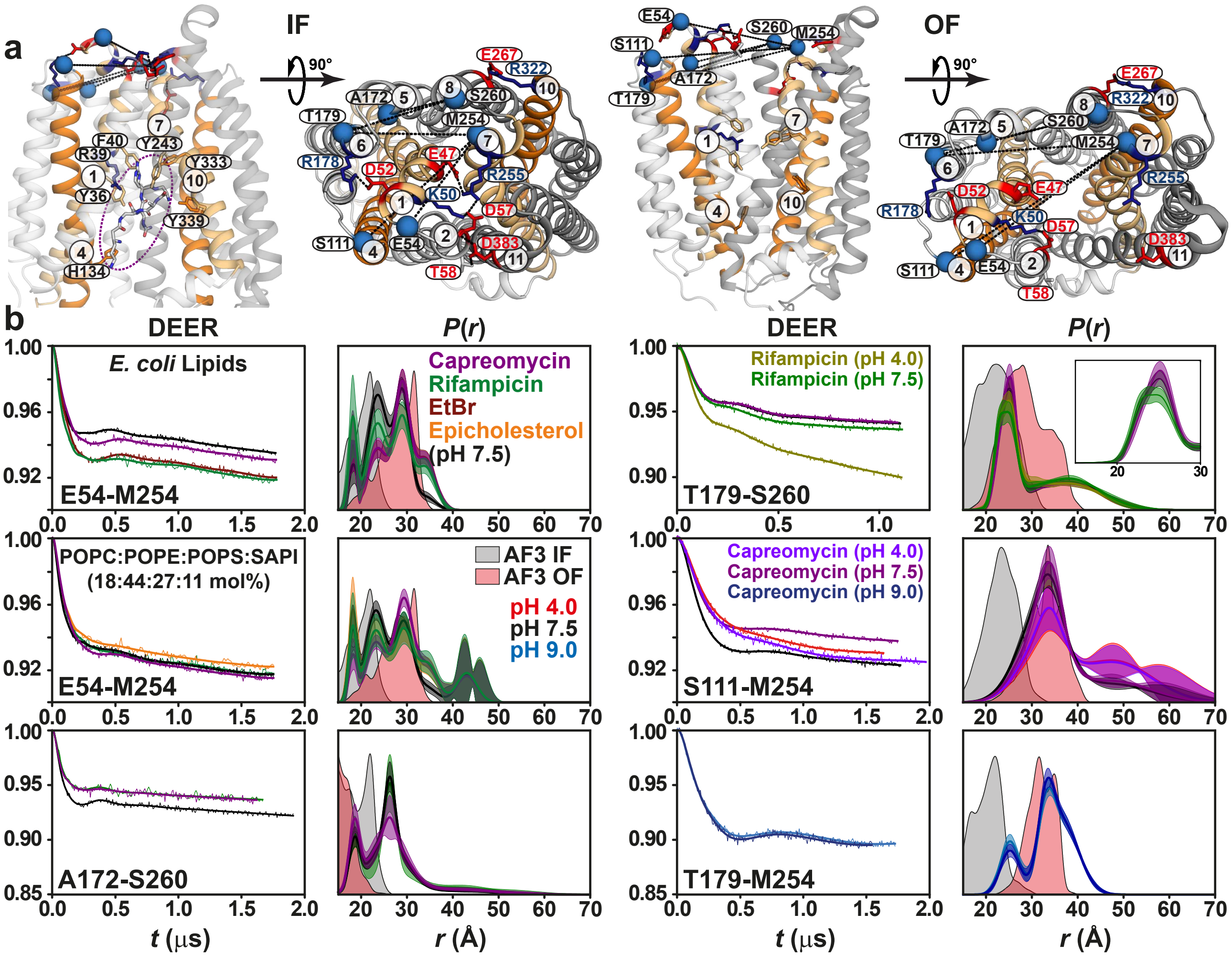

Supplementary Figure 4. Hydrophobic and hydrophilic substrates differentially shift extracellular *Ms*Spns conformational equilibria. (**a**) Membrane and periplasmic views of the IF and OF models. Capreomycin (circled) is docked in the IF substrate-binding cavity, with conserved aromatic residues in binding cavity shown as sticks and DEER spin-label pairs shown as blue spheres. Functional residue interactions stabilizing the IF conformation are highlighted in red and blue in the periplasmic views. (**b**) Raw DEER decays with fits and corresponding extracellular distance distributions measured in the apo state and in the presence of substrates. Two lipid compositions were tested for the E54–M254 pair. Water-soluble cationic substrates, including capreomycin and ethidium bromide, stabilize the OF conformation, whereas lipophilic substrates, including rifampicin and epicholesterol, shift the equilibrium toward the IF state.

bioRxiv preprint doi: https://doi.org/10.64898/2026.05.09.724020; this version posted June 1, 2026. The copyright holder for this preprint (which was not certified by peer review) is the author/funder, who has granted bioRxiv a license to display the preprint in perpetuity. It is made available under a CC-BY 4.0 International license.

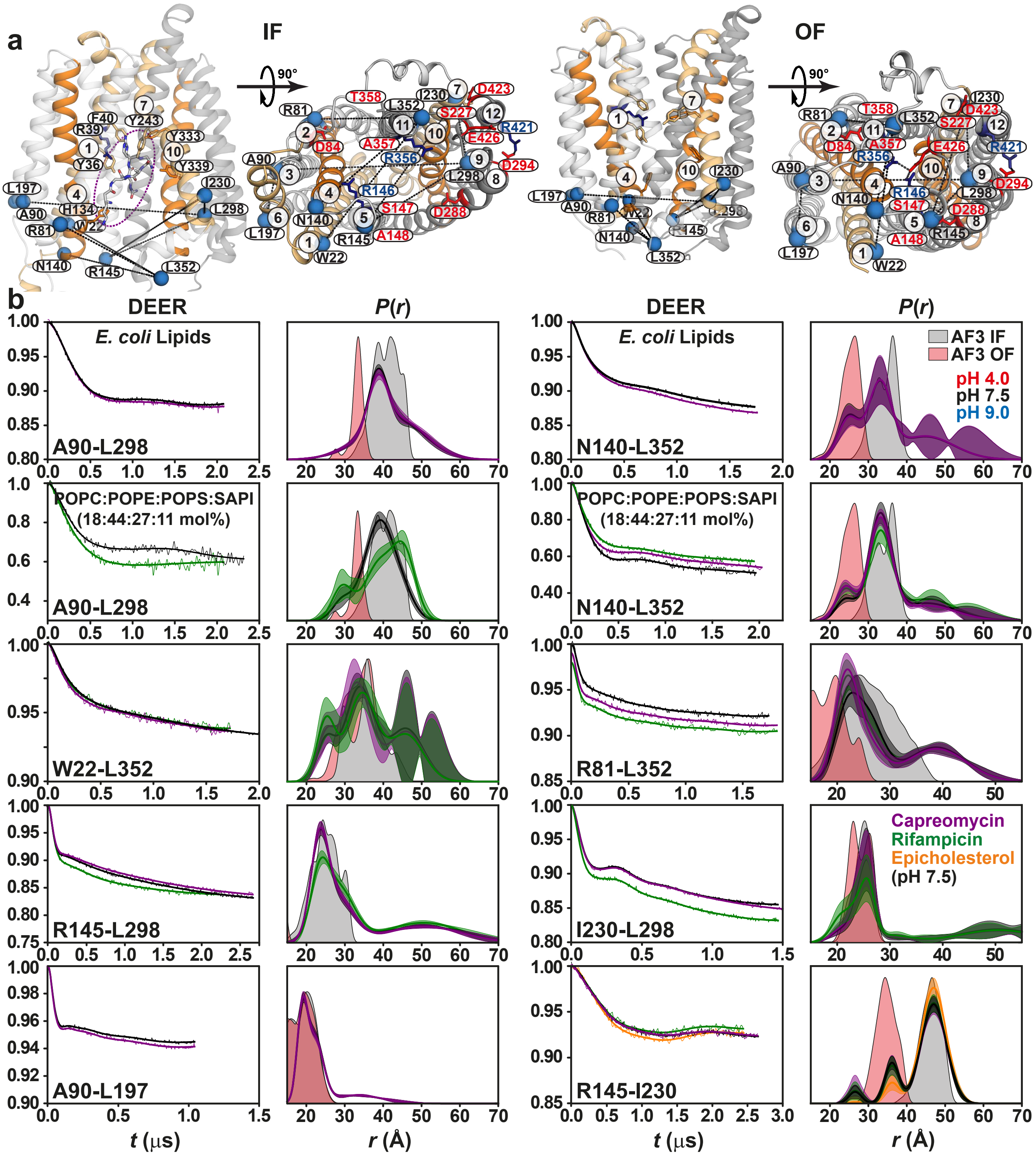

Supplementary Figure 5. Substrate hydrophobicity drives opposing shifts in intracellular *Ms*Spns conformational equilibria. (**a**) Membrane and cytoplasmic views of the IF and OF models. Capreomycin is docked in the IF substrate-binding cavity, with conserved aromatic residues shown as sticks and DEER spin-label pairs shown as blue spheres. Functional residue interactions that stabilize the OF conformation are highlighted in red and blue in the cytoplasmic views. (**b**) Raw DEER decays with fits and corresponding intracellular distance distributions measured in the apo state and in the presence of substrates. Two lipid compositions were tested for the A90–L298 and N140–L352 pairs. Water-soluble cationic substrates, including capreomycin and ethidium bromide, stabilize the OF conformation, whereas lipophilic substrates, including rifampicin and epicholesterol, shift the equilibrium toward the IF state.

bioRxiv preprint doi: https://doi.org/10.64898/2026.05.09.724020; this version posted June 1, 2026. The copyright holder for this preprint (which was not certified by peer review) is the author/funder, who has granted bioRxiv a license to display the preprint in perpetuity. It is made available under a CC-BY 4.0 International license.

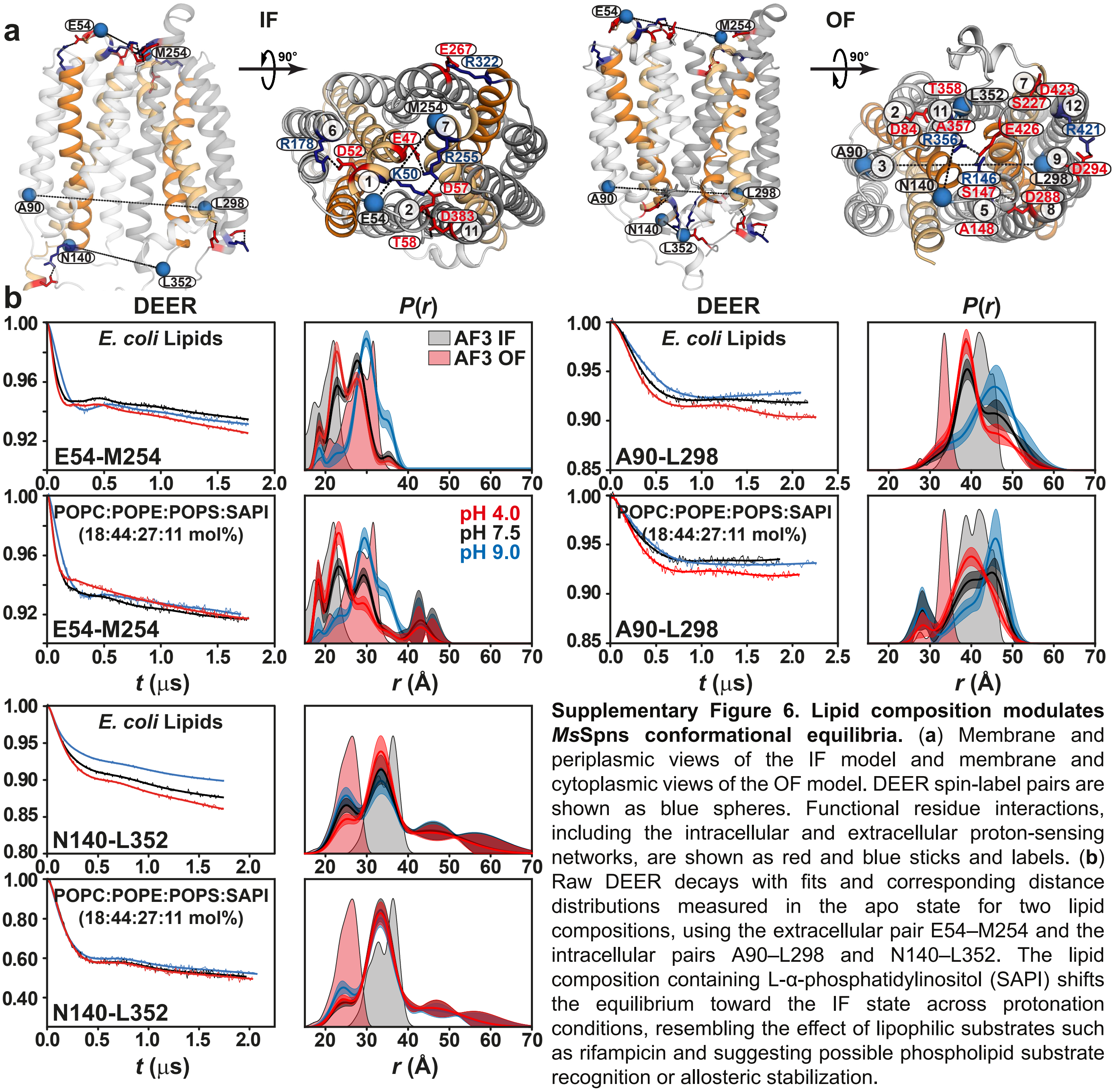

Supplementary Figure 6. Lipid composition modulates *Ms*Spns conformational equilibria. (**a**) Membrane and periplasmic views of the IF model and membrane and cytoplasmic views of the OF model. DEER spin-label pairs are shown as blue spheres. Functional residue interactions, including the intracellular and extracellular proton-sensing networks, are shown as red and blue sticks and labels. (**b**) Raw DEER decays with fits and corresponding distance distributions measured in the apo state for two lipid compositions, using the extracellular pair E54–M254 and the intracellular pairs A90–L298 and N140–L352. The lipid composition containing L-α-phosphatidylinositol (SAPI) shifts the equilibrium toward the IF state across protonation conditions, resembling the effect of lipophilic substrates such as rifampicin and suggesting possible phospholipid substrate recognition or allosteric stabilization.

bioRxiv preprint doi: https://doi.org/10.64898/2026.05.09.724020; this version posted June 1, 2026. The copyright holder for this preprint (which was not certified by peer review) is the author/funder, who has granted bioRxiv a license to display the preprint in perpetuity. It is made available under a CC-BY 4.0 International license.

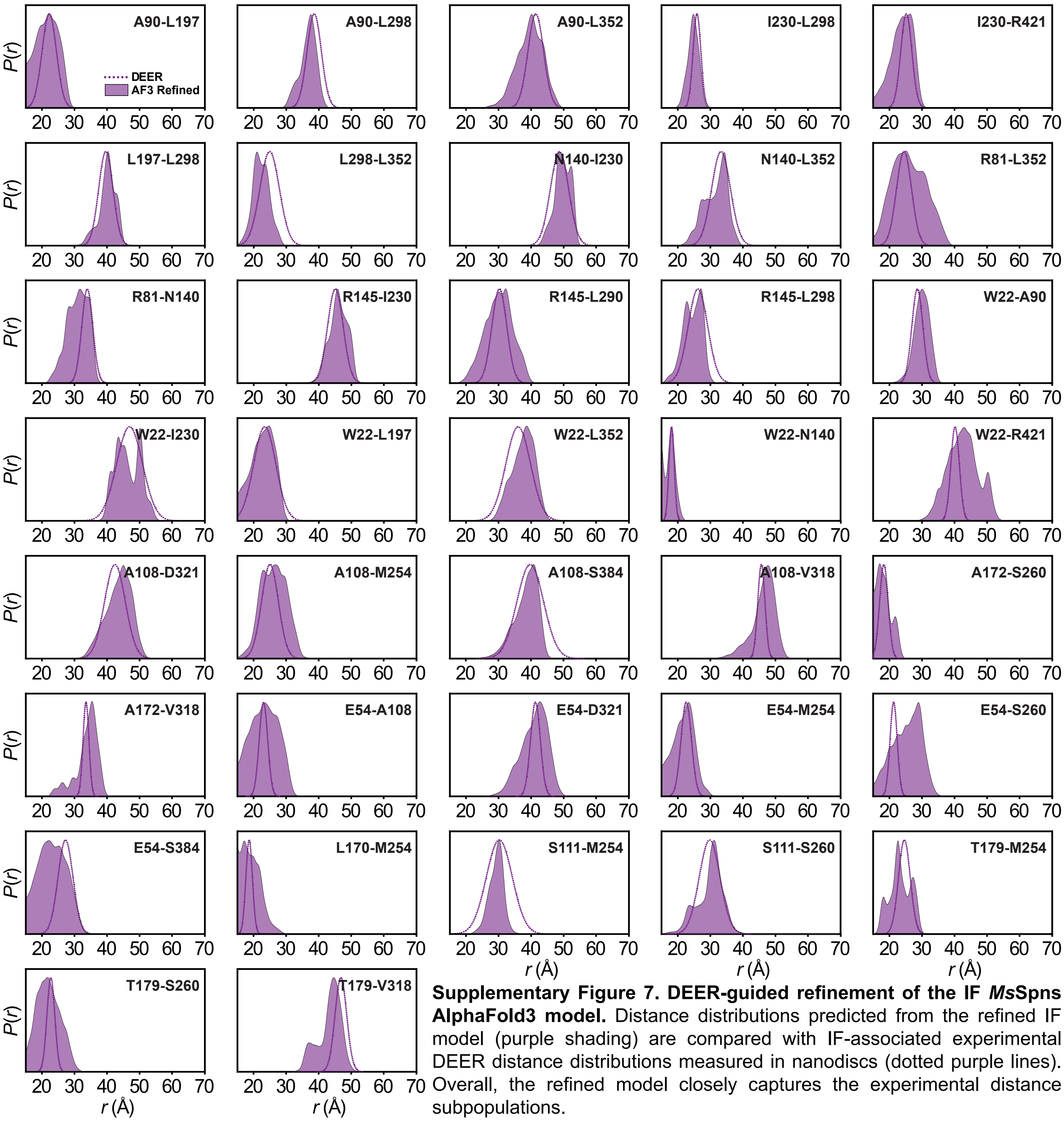

Supplementary Figure 7. DEER-guided refinement of the IF *Ms*Spns AlphaFold3 model. Distance distributions predicted from the refined IF model (purple shading) are compared with IF-associated experimental DEER distance distributions measured in nanodiscs (dotted purple lines). Overall, the refined model closely captures the experimental distance subpopulations.

bioRxiv preprint doi: https://doi.org/10.64898/2026.05.09.724020; this version posted June 1, 2026. The copyright holder for this preprint (which was not certified by peer review) is the author/funder, who has granted bioRxiv a license to display the preprint in perpetuity. It is made available under a CC-BY 4.0 International license.

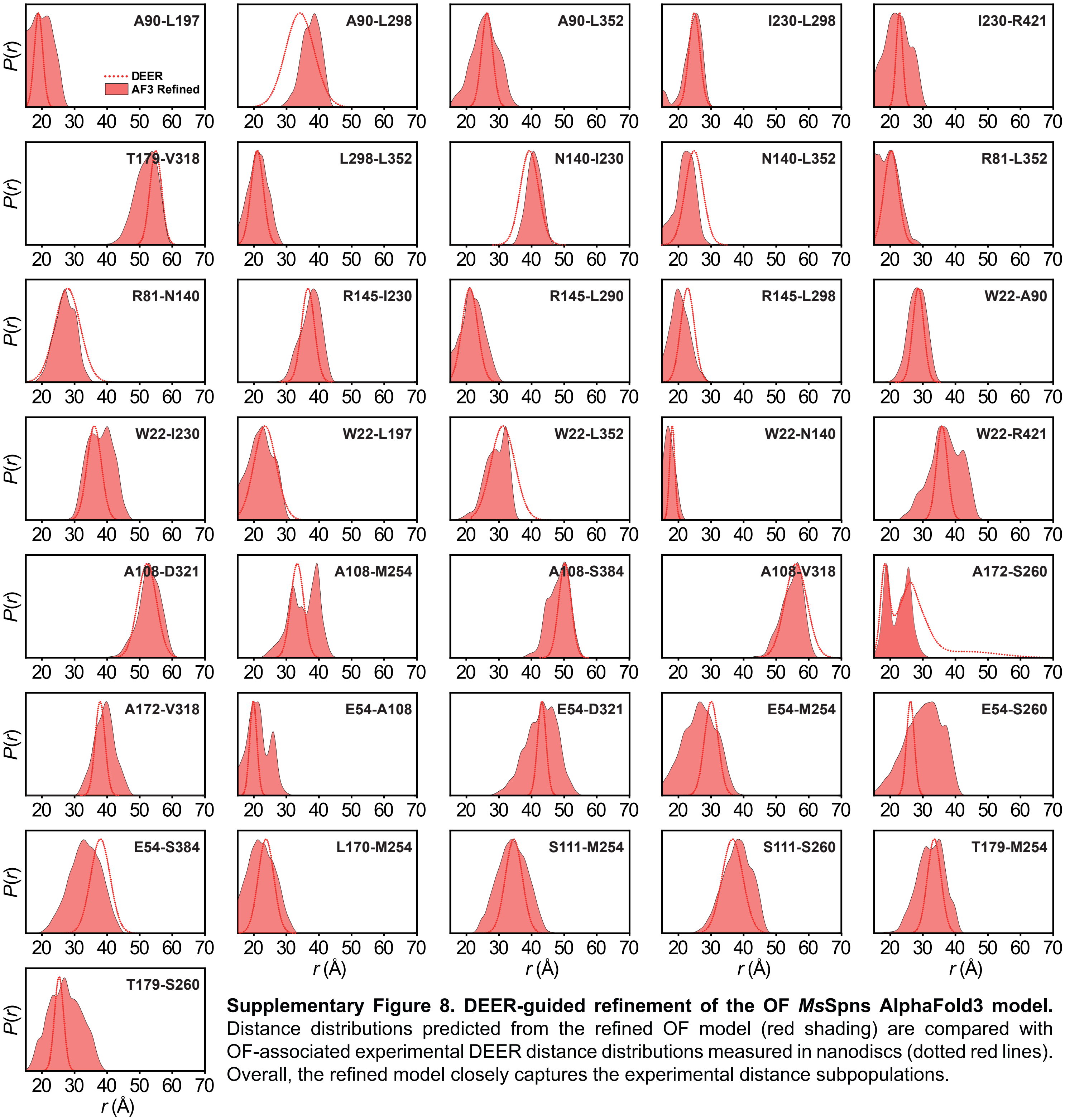

Supplementary Figure 8. DEER-guided refinement of the OF *Ms*Spns AlphaFold3 model. Distance distributions predicted from the refined OF model (red shading) are compared with OF-associated experimental DEER distance distributions measured in nanodiscs (dotted red lines). Overall, the refined model closely captures the experimental distance subpopulations.

bioRxiv preprint doi: https://doi.org/10.64898/2026.05.09.724020; this version posted June 1, 2026. The copyright holder for this preprint (which was not certified by peer review) is the author/funder, who has granted bioRxiv a license to display the preprint in perpetuity. It is made available under a CC-BY 4.0 International license.

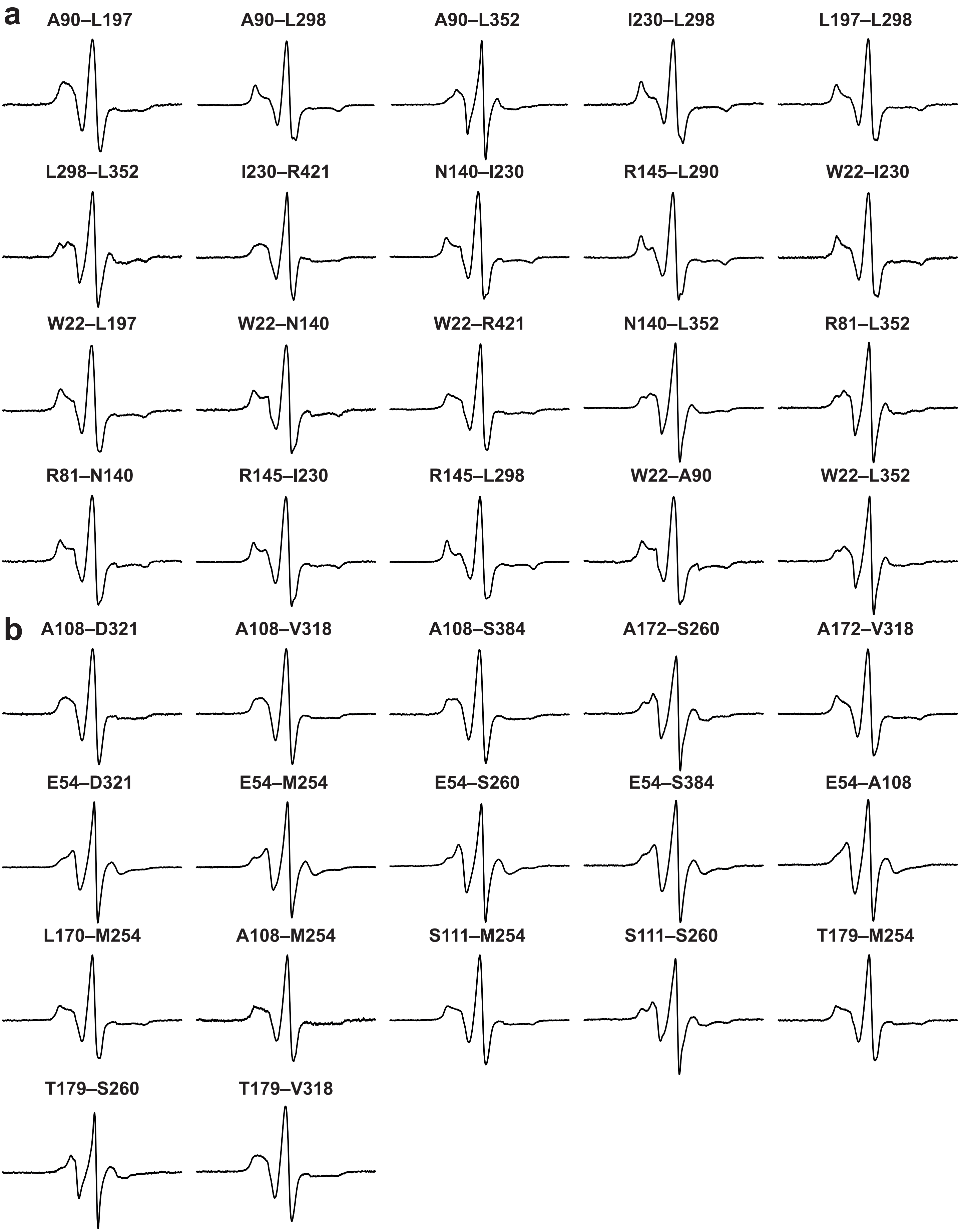

Supplementary Figure 9. CW EPR spectra of spin-labeled double-cysteine mutants for DEER spectroscopy.

bioRxiv preprint doi: https://doi.org/10.64898/2026.05.09.724020; this version posted June 1, 2026. The copyright holder for this preprint (which was not certified by peer review) is the author/funder, who has granted bioRxiv a license to display the preprint in perpetuity. It is made available under a CC-BY 4.0 International license.

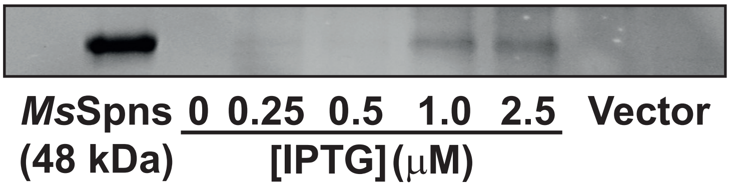

Supplementary Figure 1a

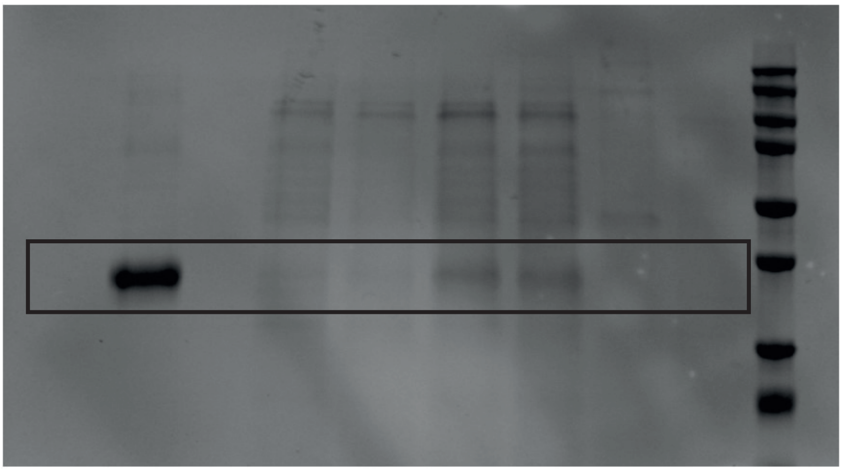

Supplement: Supplementary file 1 [file NIHPP2026.05.09.724020v2-supplement-1.pdf]
